# Maternal monosaccharide diets modulate melanocortin-4 receptor signaling and metabolic state in rat offspring

**DOI:** 10.1007/s43440-025-00785-8

**Published:** 2025-09-16

**Authors:** Kacper Witek, Karolina Wydra, Agata Suder, Małgorzata Filip

**Affiliations:** Department of Drug Addiction Pharmacology, Maj Institute of Pharmacology, Polish Academy of Sciences, Smętna 12, Kraków, PL-31-343 Poland

**Keywords:** Fructose, Glucose, Glucose homeostasis, MC4R, Metabolism

## Abstract

**Background:**

Maternal consumption of monosaccharides during pregnancy and lactation can program long-term metabolic and neurobehavioral outcomes in offspring. The melanocortin-4 receptor (MC4R) is a key regulator of metabolism and behavior. However, the impact of maternal monosaccharide diets on MC4R signaling within mesocorticolimbic regions remains unclear. In this study, we investigated the effects of maternal glucose (GLU) and fructose (FRU) diets on metabolic, molecular, and neurochemical outcomes in offspring.

**Methods:**

Adolescent and young adult male and female Wistar rat offspring, following maternal GLU and FRU exposure during pregnancy and lactation, underwent sucrose preference testing, intraperitoneal glucose tolerance tests, and serum lipid profiling. In addition, the gene expression of *Mc4r*, proopiomelanocortin (*Pomc*), agouti-related peptide (*Agrp*), and melanocortin-2 receptor accessory protein 2 (*Mrap2*) was quantified in the nucleus accumbens, prelimbic cortex, dorsal striatum, and basolateral amygdala, while the levels of MC4R protein were assessed in synaptosomal fractions from these brain regions.

**Results:**

The maternal GLU diet reduced total calorie intake during lactation, while the FRU diet increased the dams’ caloric intake from sugar during both pregnancy and lactation. In the offspring, a maternal FRU diet increased sucrose consumption in young adult males and dysregulated glucose homeostasis in both adolescent and young adult males. Maternal monosaccharide diets also influenced serum lipid profiles and increased the body weights of their offspring. At the molecular level, region-, sex-, and age-specific changes in gene expression were observed, particularly the upregulation of *Mc4r*. Neurochemical analyses showed that maternal FRU diet increased synaptosomal MC4R levels in the mesocorticolimbic regions of young adult offspring. Principal component analysis revealed distinct clustering of metabolic and MC4R-related variables based on diet and sex, while regression analyses indicated significant diet-dependent correlations between MC4R levels and lipid parameters.

**Conclusions:**

These findings suggest that maternal monosaccharide diets induce persistent alterations in the metabolic profiles of offspring and MC4R signaling, potentially contributing to the development of programmed metabolic and behavioral outcomes.

**Clinical trial number:**

Not applicable.

**Supplementary Information:**

The online version contains supplementary material available at 10.1007/s43440-025-00785-8.

## Introduction

Maternal nutrition is an important environmental factor that plays a crucial role in fetal development and exerts long-term effects on the health of offspring [1, 2]. Among the various dietary components, the widespread production and consumption of processed foods enriched with added or artificial sugars, such as fructose found in high-fructose corn syrup (HFCS) and glucose found in sweetened beverages, contribute to unhealthy dietary patterns [3]. We recently reported that maternal or postnatal sugar exposure predisposes rodent offspring to several behavioral disturbances [4]. Specifically, a maternal fructose diet (FRU) has been shown to impair learning and memory, increase anxiety- and depressive-like behaviors, and reduce social interactions in offspring [5–9]. Our long-term experiments further supported that such monosaccharide-based maternal diets exacerbate anxiety- and depressive-like traits [10]. These findings are consistent with preclinical evidence showing that excessive sugar consumption during pregnancy and lactation can adversely affect the health and neurobehavioral development of offspring [11–13]. In humans, meta-analyses have linked high sugar intake to an increased risk of overweight or obesity [14–16], cardiovascular disease [17, 18], neurobehavioral disorders [19, 20], and type 2 diabetes [21] in both adults and children. Moreover, several large-scale birth cohort studies have associated elevated maternal consumption of sugars—including fructose—during pregnancy with a greater incidence of neurodevelopmental impairments in offspring, such as attention-deficit hyperactivity disorder, autism spectrum disorder, externalizing behaviors, and cognitive deficits [22–31].

Although glucose and fructose can cross the placenta [32], their metabolism differs significantly, influencing offspring development. During gestation, glucose is the primary energy substrate for the fetus, actively transported across the placenta via glucose transporters (GLUTs) [33]. Furthermore, glucose is transported in the small intestine via sodium-glucose-linked transporters, which stimulate insulin and leptin secretion. In contrast, fructose is passively absorbed via the fructose-specific transporter GLUT5 and primarily metabolized in the liver. Fructose enters glycolytic metabolism downstream of phosphofructokinase, bypassing a key regulatory step, which can promote its rapid conversion into lipid precursors through de novo lipogenesis [34]. Moreover, unlike glucose, fructose does not directly stimulate insulin or leptin release, nor does it suppress ghrelin levels to the same extent as glucose [35–37]. In several rodent studies, a maternal GLU or FRU diet induced metabolic changes in offspring, including increased levels of glucose, insulin, and leptin, as well as dysregulation of plasma cholesterol and triglyceride concentrations [38–40]. Glucose and fructose can be transported across the blood-brain barrier by GLUTs, directly or indirectly affecting the central nervous system through the modulation of metabolic and hormonal signals [41, 42]. More specifically, maternal or early-life exposure to glucose or fructose affects microglial function [43], neuroinflammation [44, 45], hippocampal neuron degeneration [6], and plasticity [46].

Given the evidence that maternal monosaccharide intake disrupts metabolic and neurobehavioral outcomes in offspring, it is essential to identify the molecular pathways that integrate these systems. The melanocortin system plays a central role in regulating energy expenditure, feeding behavior, metabolism, and emotional processes; a key component of this system is the melanocortin-4 receptor (MC4R) [47, 48]. This G protein-coupled receptor is encoded by the *Mc4r* gene in rats. Increasing evidence indicates that *Mc4r* is highly expressed in the hypothalamus as well as in the nucleus accumbens (NAc), prelimbic cortex (PL), dorsal striatum (dSTR), and basolateral amygdala (BLA), which are involved in motivation, emotion, and reward processing [49–51]. MC4R activity is regulated by the balance between two endogenous ligands: α-melanocyte-stimulating hormone, derived from proopiomelanocortin (POMC), which activates MC4R and promotes satiety, and agouti-related peptide (AGRP), which inhibits MC4R by acting as a competitive antagonist and an inverse agonist, thus stimulating hunger [52]. This system is further modulated by peripheral hormones such as leptin (and, to a lesser extent, insulin), which enhance *Pomc* expression and suppress *Agrp* expression, thereby promoting satiety [53, 54]. In contrast, ghrelin has the opposite effect: it increases *Agrp* expression and inhibits POMC activity [55]. Additionally, the MC4R function is regulated by melanocortin-2 receptor accessory protein 2 (MRAP2), which influences receptor trafficking, surface expression, and ligand sensitivity [56]. Loss-of-function mutations in the *Mc4r* [57–59], *Pomc* [60, 61], or *Mrap2* [62] genes lead to marked hyperphagia, early-onset obesity, and enhanced somatic growth in rodent models. Conversely, restoring MC4R expression in the paraventricular nucleus of MC4R-null mice has been demonstrated to increase energy expenditure and reduce body weight, glucose levels, and insulin levels [63]. Moreover, pharmacological activation of MC4R with setmelanotide induces significant weight loss and reductions in fasting insulin and triglyceride levels [64].

Thus, this study aimed to examine the effects of maternal monosaccharide diets (GLU or FRU) during pregnancy and lactation on sucrose intake, glucose homeostasis, and serum metabolic parameters in both adolescent and young adult offspring. We also examined MC4R protein levels in synaptosomal fractions from the NAc, PL, dSTR, and BLA, along with the expression of *Mc4r* and related genes (*Pomc*, *Agrp*, *Mrap2*). Relationships between MC4R levels and metabolic outcomes were explored through multivariate and regression analyses. While the effects of maternal diets on MC4R signaling in mesocorticolimbic regions have remained largely unknown, this study aims to address this gap in the literature.

## Materials and methods

### Experimental animals and maternal diets

**Fig. 1:**
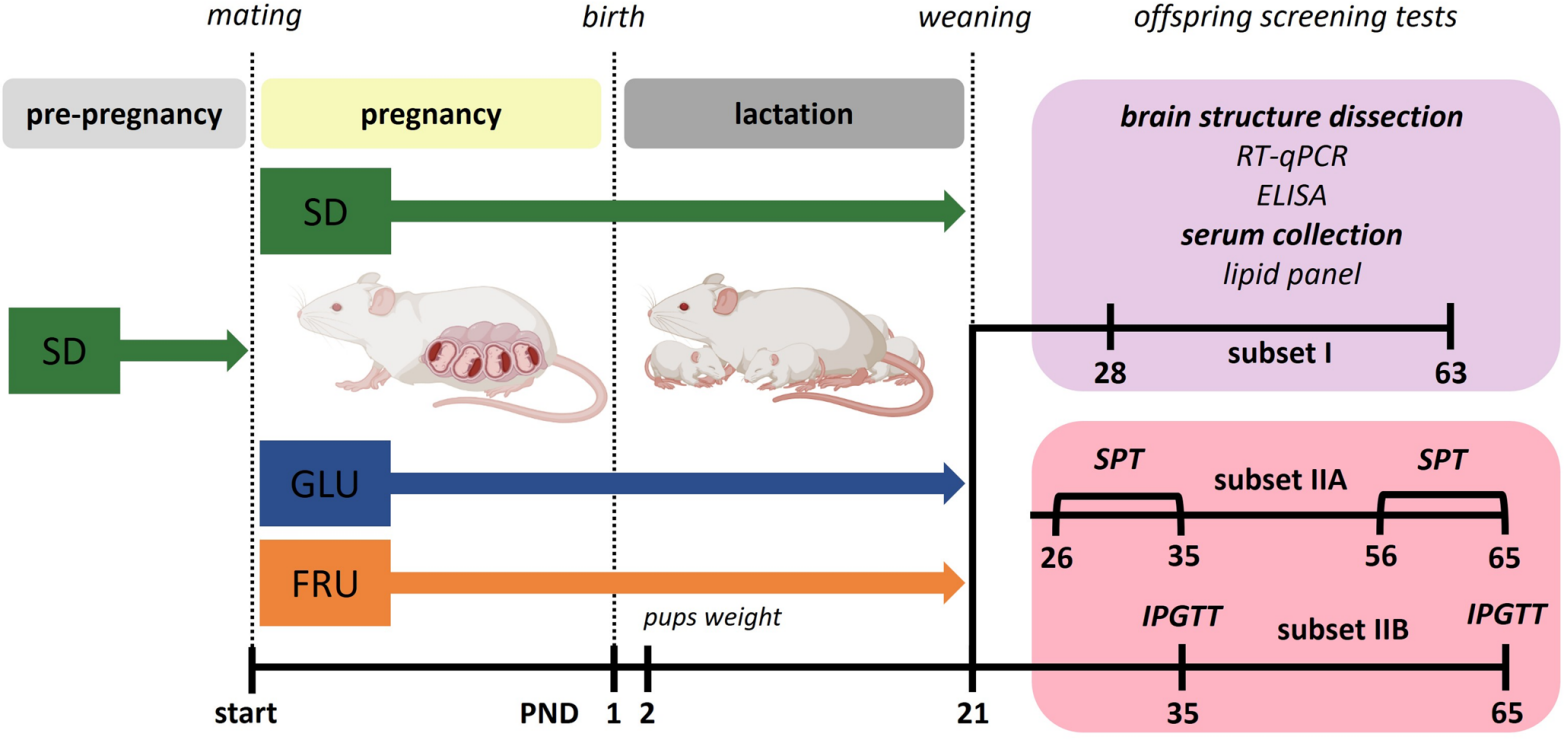
Scheme of the experiment. Dams before mating (pre-pregnancy) were fed a control diet (SD), while during pregnancy (~ 21 days) and lactation (~ 21 days), they were fed one of three diets: control (SD), glucose (GLU), or fructose (FRU). After weaning at postnatal day 21 (PND21), the offspring from each maternal diet group was divided into two main subsets: the first subset (subset I) was used for molecular analyses (RT-qPCR), neurochemical analyses (ELISA), and the serum lipid panel; the second subset (subset II) was further divided into subset IIA for the sucrose preference test (SPT) and subset IIB for the intraperitoneal glucose tolerance test (IPGTT). All pups were fed SD chow after weaning

We used Wistar rats derived from the licensed Charles River breeder (Sulzfeld, Germany), which were housed in standard transparent plastic rodent cages in an animal breeding room at 21 ± 2 °C and 40 ± 10% humidity, with a 12-hour light–dark (LD 12:12 h) cycle (lights on at 6:00 a.m.). Animals had free access to water and *ad libitum* standard VRF1 (P) rodent food (Special Diets Services, London, UK; Cat. No. 801900). To re-evaluate the influence of the maternal monosaccharide diet on offspring, we implemented the same procedures used in our long-term study [10]. Thus, twenty-four virgin females (201–225 g), after the acclimatization period (14 days) and during the proestrus phase (the estrous cycle phases, determined by daily vaginal smears), were mated with six randomly assigned males (226–250 g) until sperm presence was confirmed in the smears. Pregnant females (8 dams per group) were individually housed and randomly assigned to one of three diet groups (Table 1): the standard VRF1 (P) diet (hereafter referred to as SD), or one of two custom-formulated synthetic diets modeled on VRF1, in which starch–sucrose was selectively replaced with either glucose (GLU) or fructose (FRU), as previously described [10]. During pregnancy (21 ± 2 days) and lactation (21 ± 2 days), dams were given *ad libitum* access to water and solid food (administered by feeder), according to the diet group (Fig. 1). For each maternal diet (SD, GLU, FRU), one cohort of offspring was obtained from eight different dams mated with six different males to reduce the influence of parental, genetic, and individual predispositions [65].

**Table 1:** The composition of the main macronutrients, expressed as a percentage of energy, and the energy value of the maternal experimental diets during pregnancy and lactation

Diet		SD	GLU	FRU
Carbohydrates	Pectin (%)	1.37	0.00	0.00
	Hemicellulose (%)	8.76	0.13	0.13
	Cellulose (%)	3.95	6.40	6.21
	Lignin (%)	1.06	0.00	0.00
	**Starch (%)**	**35.41**	**0.00**	**0.00**
	**Sugar (%)**	**4.64 ** **(from sucrose)**	**53.76 ** **(from glucose)**	**54.35** **(from fructose)**
Protein (%)		19.11	19.13	18.56
Fat (%)		4.75	5.06	4.91
Nitrogen Free Extract (%)		56.10	56.02	57.03
Crude Fibre (%)		3.85	4.55	4.42
Total energy (kcal/g)		3.40	3.44	3.43

The glucose (GLU) and fructose (FRU) diets are synthetic analogues of the standard VRF1 (P) diet (SD), with the type of sugar specifically modified to match the SD diet

On postnatal day 2 (PND2), litter size was recorded, and mean pup body weight per litter was measured. A total of 211 offspring were generated: 74 SD, 67 GLU, and 70 FRU. At PND2, litters were standardized to 10 pups when possible, resulting in the exclusion of 16 animals: 7 SD, 4 GLU, and 5 FRU. During weaning (at PND21), the offspring were weaned, separated by sex, individually marked, and transitioned to an SD; a further 9 animals were excluded as outliers based on post-weaning body weight (3 SD, 5 GLU, and 1 FRU). After exclusions, a total of 186 animals were retained for analysis (64 SD, 58 GLU, and 64 FRU). Offspring from each maternal diet group were randomly allocated (using a computer-generated randomization schedule) into two main subsets: subset I for molecular (RT-qPCR), neurochemical (ELISA), and serum lipid analyses (*n* = 8 per group); and subset II, which was further divided into subset IIA for the sucrose preference test (SPT) and subset IIB for the intraperitoneal glucose tolerance test (IPGTT). Sample sizes for the SPT and IPGTT were dictated by the number of offspring available after litter size standardization and exclusion of post-weaning outliers. Offspring were housed in standard transparent plastic rodent cages, either in groups of five per cage (38 × 59 × 20 cm) or individually (26.5 × 42 × 18 cm) during the SPT and IPGTT. Due to the use of diet-based animal marking, blinding was not possible during the SPT and the IPGTT. The outcome assessment and data analysis were conducted while blinded to group allocation. All experiments were carried out in conformity with the European Union Directive (2010/63/EU), the Polish Act on the Protection of Animals (Dz.U. z 2020 r. poz. 638), and with the approval of the II Local Ethics Committee for Animal Experiments at the Maj Institute of Pharmacology, Polish Academy of Science, Kraków, Poland (approval numbers 18/2021 and 54/2021) under the three Rs rule.

### Sucrose preference test (SPT)

The SPT is a reward-based assay used to assess anhedonia. In this study, we tested a subset IIA of male (6 per group) and female (8 SD, 7 GLU, and 10 FRU) adolescent offspring (at PND35) and retested them in young adulthood (at PND65) to evaluate stress-related responses and sucrose consumption. Before testing, the rats were acclimatized to a single drinking water bottle in their home cages for at least four days, starting at PND26 and PND56, respectively. Following acclimatization, the rats were given access to two water bottles in their home cages for an additional two days. Subsequently, they were given access to two bottles: one containing tap water and the other containing a 1% sucrose solution (Sigma-Aldrich, Merck, Darmstadt, Germany; Cat. No. S9378) for an additional two days. To standardize conditions, all rats underwent 12 h of food and water deprivation before the test. Following this fasting period, water and sucrose solution consumption were measured over a 1-hour test session. During the experiment, rats were kept individually in a cage, and body weight, water intake, and sucrose intake were recorded daily throughout the study. To prevent potential side bias, the positions of the bottles were rotated between the left and right sides. Sucrose preference was calculated for the test session (Test) and the two days preceding the test deprivation (Baseline). The sucrose preference percentage was determined using the following formula:

$$\begin{gathered} \% \,\operatorname{Preference} \, = \,(({\text{sucrose}}\,\operatorname{intake} \, \hfill \\ \,/\,({\text{sucrose}}\,\operatorname{intake} \, + \,{\text{water}}\,\operatorname{intake} ))\, \times\,100 \hfill \\ \end{gathered} $$

### Metabolic and biochemical procedures

#### Intraperitoneal glucose tolerance test (IPGTT)

To evaluate glucose metabolism and tolerance, the IPGTT was performed in a subset IIB of adolescents (at PND35) and retested in young adults (at PND65) males (7 SD, 6 GLU, and 7 FRU) and females (11 SD, 7 GLU, and 9 FRU). Briefly, basal blood glucose levels (mg/dL) were measured from tail-vein samples using a Contour Plus glucometer (Bayer, Leverkusen, Germany) following an 8-hour fasting period. Next, a 20% glucose solution (Glucosum anhydricum; Hasco-Lek S.A., Wrocław, Poland), prepared in physiological saline (0.9% sodium chloride solution; Polpharma S.A., Starogard Gdański, Poland), was administered intraperitoneally (*i.p.*) at a volume (mL) equivalent to 10% of the rat’s body weight (g). Following glucose administration, blood glucose levels were measured at 15, 30, 60, 90, and 120 min post-injection. Blood glucose concentrations (mg/dL) were converted to (mmol/L), and glucose tolerance was assessed by calculating the total glucose excursion as the area under the curve (AUC). During the experiment, each rat was housed individually within a cage.

#### Serum collection and metabolic parameters analyses

Peripheral blood samples were collected from naïve adolescent (at PND28) and young adult (at PND63) offspring from subset I (8 per group) during decapitation. The clotted blood was centrifuged for 20 min at 4 °C at 3,500 rpm, after which the serum fraction was carefully transferred into Eppendorf tubes and stored at -80 °C until further analyses. Serum glucose levels and lipid panels, including low-density lipoprotein (LDL), high-density lipoprotein (HDL), total cholesterol, and triglycerides, were analyzed by a specialist veterinary diagnostic laboratory (Lab-Wet, Kraków, Poland) using the BS-800 M modular system analyzer (Mindray Medical Poland, Warsaw, Poland). HDL and LDL levels were measured directly and expressed in mg/dL. Total cholesterol, triglycerides, and glucose levels (mg/dL) were determined using colourimetric and enzymatic assays, including cholesterol esterase and cholesterol oxidase (CHOD/PAP) methods for total cholesterol, glycerophosphate oxidase method for triglycerides, and glucose oxidase method for glucose.

### Molecular and neurochemical procedures

#### Brain structures collection

Naïve adolescent (PND28) and young adult (PND63) offspring from subset I (*n* = 8 per group) were sacrificed without anaesthesia by decapitation, 3 h after lights-on in the housing room. Brain structures were dissected under cold conditions using razor blades. Target regions were collected as close as possible to the Bregma-referenced coordinates (in mm) for the anterior–posterior (AP), medial–lateral (ML), and dorsal–ventral (DV) axes from the Rat Brain Atlas [66], guided by histological landmarks: the basolateral amygdala (BLA: AP ~ -1.8 to -3.3 mm; ML ~ 4.0 to 5.0 mm; DV ~ 7.8 to 9.0 mm), dorsal striatum (dSTR: AP ~ 1.7 to -0.4 mm; ML ~ 1 ± 3.8 mm; DV ~ 3.4 to 6.0 mm), nucleus accumbens (NAc: AP ~ 1.7 to 0.7 mm; ML ~ 0.2 to 2.6 mm; DV ~ 6.0 to 8.4 mm), and prelimbic cortex (PL: AP ~ 3.2 to 2.2 mm; ML ~ 0 ± 1.4 mm; DV ~ 3.0 to 4.6 mm). Immediately after dissection, brain structures were snap-frozen on dry ice and stored at -80 °C until further analysis.

#### Synaptosomal fraction preparation and MC4R protein levels

The MC4R protein level was measured in the synaptosomal fraction following the synaptosomal preparation protocol described by Kamat et al. [67]. Briefly, brain tissue was homogenized using an EpiShear™ Probe Sonicator, Model CL-18 (Active Motif, Carlsbad, CA, USA) in 10% (w/v) of 0.32 M sucrose (Sigma-Aldrich, Merck, Darmstadt, Germany; Cat. No. S9378) dissolved in HEPES (BioShop Canada Inc., Burlington, ON, Canada; Cat. No. HEP001) buffer (pH 7.4), supplemented with a cOmplete™ (Protease Inhibitor Cocktail, Roche Diagnostics GmbH, Mannheim, Germany; Cat. No. 11697498001). The homogenate was centrifuged at 4 °C for 10 min at 600 × g. The resulting supernatant was diluted 1:1 with 1.3 M sucrose HEPES buffer to obtain a final sucrose concentration of 0.8 M. The suspension underwent two sequential washes with HEPES buffer, followed by centrifugation at 12,000 × g for 15 min at 4 °C. After each centrifugation, the supernatant was discarded, and the pellet was resuspended in RIPA Buffer (Sigma-Aldrich, Merck KGaA, Darmstadt, Germany; Cat. No. R0278), supplemented with a cOmplete™, phenylmethanesulfonyl fluoride (Sigma-Aldrich, Merck KGaA, Darmstadt, Germany; Cat. No. P7626), and 0.2% Triton™ X-100 (Sigma-Aldrich, Merck KGaA, Darmstadt, Germany; Cat. No. X100). The final centrifugation was performed at 20,000 × g for 30 min at 4 °C. The resulting synaptosomal suspension was stored at -80 °C until further analysis. Protein concentrations were determined using the Pierce™ BCA Protein Assay Kit (Thermo Fisher Scientific, Waltham, MA, USA; Cat. No. 23225) at a wavelength of 562 nm, measured with a Synergy™ HTX Multi-Mode Microplate Reader (BioTek Instruments, Winooski, VT, USA). Following the manufacturer’s protocol, the MC4R levels in the synaptosomal fractions of PL, NAc, dSTR, and BLA were quantified using ELISA kits (BT LAB, Zhejiang, China; Cat. No. E3305Ra). Briefly, each sample (40 µL) was applied in duplicate along with MC4R standards (0, 0.0375, 0.075, 0.15, 0.30, 0.60, 1.2, and 2.4 ng/mL) to 96-well ELISA plates. The absorbance was measured at 450 nm using a Synergy™ HTX Multi-Mode Microplate Reader, and the MC4R concentration was calculated from a standard curve, expressed as ng/mg of protein.

#### RNA extraction and quantitative real-time PCR (RT-qPCR) analyses

Total RNA from collected tissue was extracted using the Bead-Beat Total RNA Mini Kit (A&A Biotechnology, Gdynia, Poland; Cat. No. K-PKCM-100) according to the manufacturer’s protocol. Briefly, frozen tissues were homogenized using a TissueLyser II (Qiagen, Aosheng, Hangzhou, China) in the presence of a phenazone solution. The homogenate was then mixed with chloroform and centrifuged. The resulting supernatant was combined with isopropanol, transferred to a minicolumn for RNA purification, and subjected to triple washing with A1 washing solution, followed by centrifugation. The total RNA concentration was measured using a NanoDrop^®^ ND-1000 Spectrophotometer (NanoDrop Technologies Inc., Wilmington, DE, USA), with sample purity assessed by the absorbance ratio at 260 and 280 nm. Complementary DNA (cDNA) was synthesized from total RNA using the High-Capacity RNA-to-cDNA™ Kit (Applied Biosystems™, Thermo Fisher Scientific, Vilnius, Lithuania; Cat. No. 4368813) in a T100™ Thermal Cycler (Bio-Rad Laboratories Inc., California, USA). Quantitative real-time PCR (RT-qPCR) was performed using the QuantStudio™ 3 (Applied Biosystems™, Thermo Fisher Scientific, Foster City, CA, USA), TaqMan^®^ Gene Expression Master Mix (Applied Biosystems™, Vilnius, Lithuania; Cat. No. 4369016) and TaqMan^®^ Gene Expression Assays (Applied Biosystems™) for the following target genes: *Agrp* (ID: Rn01431703_g1), *Mc4r* (ID: Rn07311184_s1), *Mrap2* (ID: Rn01445736_m1) and *Pomc* (ID: Rn00595020_m1). The PCR cycling conditions were as follows: initial denaturation at 95 °C for 10 min, followed by 40 cycles of 95 °C for 15 s and 60 °C for 60 s. Gene expression levels were analyzed using the comparative CT method (2^^−ΔΔCT^) [68] and normalized to the housekeeping gene hypoxanthine phosphoribosyltransferase 1 (*Hprt1*, ID: Rn01527840_m1). Results are expressed as fold change relative to the SD group.

### Statistical analyses

Experimental data are expressed as the mean ± SEM (standard error of the mean). Statistical analyses were performed with Statistica 13.3 (TIBCO Software Inc., Palo Alto, CA, USA) and visualized in GraphPad Prism 10.0.0 (GraphPad Software, Inc., San Diego, CA, USA). The Shapiro-Wilk test and the Levene test were used to test the normality and homogeneity of variance, respectively. For normally distributed data, one-way and two-way analyses of variance (ANOVA; F-statistic) were used to assess differences between the experimental groups. Alternatively, for time- or age-dependent data, two-way repeated measures ANOVA (F statistic) with or without Geisser-Greenhouse correction was performed. For non-normally distributed data, a nonparametric Kruskal-Wallis test by ranks (H-statistic) was performed. For statistical differences between groups, either parametric Tukey’s and Dunnett’s or nonparametric Dunn’s multiple comparisons post hoc tests were performed. N corresponds to the number of individuals. A p-value of less than 0.05 was considered statistically significant.

Molecular and neurochemical analyses were conducted in Python (Python Software Foundation, version 3.13). For gene expression and protein levels, three-way ANOVAs (F-statistic) with partial eta-squared ($\:{\eta\:}_{p}^{2}$) as the effect size were performed, followed by Tukey’s multiple comparisons post hoc tests. Principal component analysis (PCA) was conducted separately for adolescent (PND28) and young adult (PND63) offspring on standardized metabolic parameters (body weight, glucose, lipid profile) and synaptosomal MC4R protein levels in four brain regions (BLA, NAc, dSTR, PL). Suitability for PCA was assessed using Bartlett’s test of sphericity and the Kaiser–Meyer–Olkin (KMO) measure. The first two principal components (PC1 and PC2) were retained, and variables with absolute loadings > 0.3 were selected for further analyses. Multivariate analysis of variance (MANOVA) tested the effects of maternal diet, sex, and their interaction on PC1 and PC2 scores. Subsequently, two-way ANOVAs were performed for each principal component. For significant ANOVA effects, post hoc comparisons were conducted using Tukey’s HSD test, where the assumptions of normality (Shapiro–Wilk) and homogeneity of variance (Levene’s test) were met; otherwise, Dunn’s test with Bonferroni correction was used. False discovery rate (FDR) correction was applied to p-values from ANOVAs and post hoc tests. Effect sizes were reported as $\:{\eta\:}_{p}^{2}$. Multiple linear regressions were performed separately for each combination of age, sex, MC4R region, and metabolic parameter. Models included main effects of MC4R levels, diet group (SD, GLU, FRU), and interactions between MC4R levels and diet groups. Diagnostic checks included Shapiro–Wilk tests for residual normality and Breusch–Pagan tests for heteroskedasticity. Cohen’s f² was calculated as an effect size for the regression models. Significant regression findings were visualized with scatter plots, fitted regression lines, and confidence intervals. Additionally, Spearman correlation coefficients (ρ) were computed and shown on plots to illustrate the strength and direction of bivariate relationships. A significance threshold of *p* < 0.05 was applied throughout the analyses.

## Results

### Effects of maternal monosaccharide diets on dams’ body weight and caloric intake

**Fig. 2:**
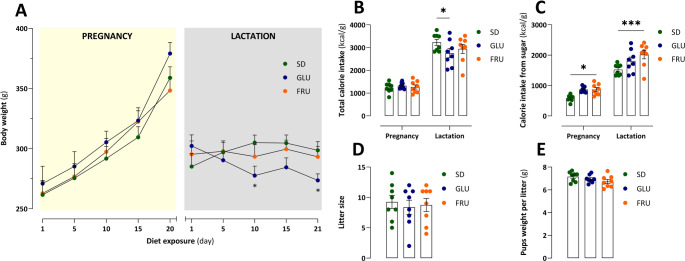
Effects of maternal glucose (GLU) or fructose (FRU) diet intake on dam body weight and calorie intake during pregnancy and lactation, as well as on litter size and pup weight. (**A**) Dams’ body weight (g) during pregnancy and lactation. (**B**) Total calorie intake (kcal/g) during pregnancy and lactation. (**C**) Calorie intake from sugar (kcal/g) during pregnancy and lactation. *n* = 8 dams per group. Data are analyzed by two-way repeated measure ANOVA with or without Geisser-Greenhouse’s correction, followed by Dunnett’s multiple comparison test. (**D**) Litter size of the dams. (**E**) Pups’ weight (g) per litter. *n* = 8 dams per group. Data are analyzed by one-way ANOVA. Data are compared to the control diet (SD) groups (*p < 0.05, ***p < 0.001)

No diet effect (F_2, 21_ = 0.001, *p* = 0.099) was observed; however, significant interaction (F_18, 189_ = 5.07, *p* < 0.0001) and time (F_1.294, 27.17_ = 96.11, *p* < 0.0001) effects were noted regarding the body weight of dams during pregnancy and lactation. During lactation, only GLU dams exhibited body weights that were 9% and 8% lower than those of SD dams on days 10 and 21 (Fig. 2A). Based on diet caloricity (Table 1), no diet (F_2, 21_ = 0.80, *p* = 0.46) or interaction (F_2, 21_ = 2.82, *p* = 0.08) effects were detected regarding total calorie intake. Nevertheless, the time effect (F_1, 21_ = 305.90, *p* < 0.0001) indicated that only GLU dams decreased their total calorie consumption during lactation by 14% compared to SD dams, with no changes observed during pregnancy (Fig. 2B). Furthermore, no interaction effect (F_2, 21_ = 1.23, *p* = 0.31) was observed; however, significant effects of time (F_1, 21_ = 258.10, *p* < 0.0001) and diet (F_2, 21_ = 8.41, *p* = 0.002) were noted in calorie intake from sugar. During the pregnancy and lactation periods, FRU dams displayed a 49% and 32% higher calorie intake from sugar compared to SD dams, respectively (Fig. 2C). The diet effect (F_2, 21_ = 0.15, *p* = 0.86) did not influence litter size; however, disparities in litter sizes among the dams were observed (Fig. 2D). Additionally, maternal diet did not affect the pups weight (F_2, 21_ = 1.85, *p* = 0.18) measured per litter (Fig. 2E).

### Effects of maternal monosaccharide diets on offspring sucrose preference

**Fig. 3:**
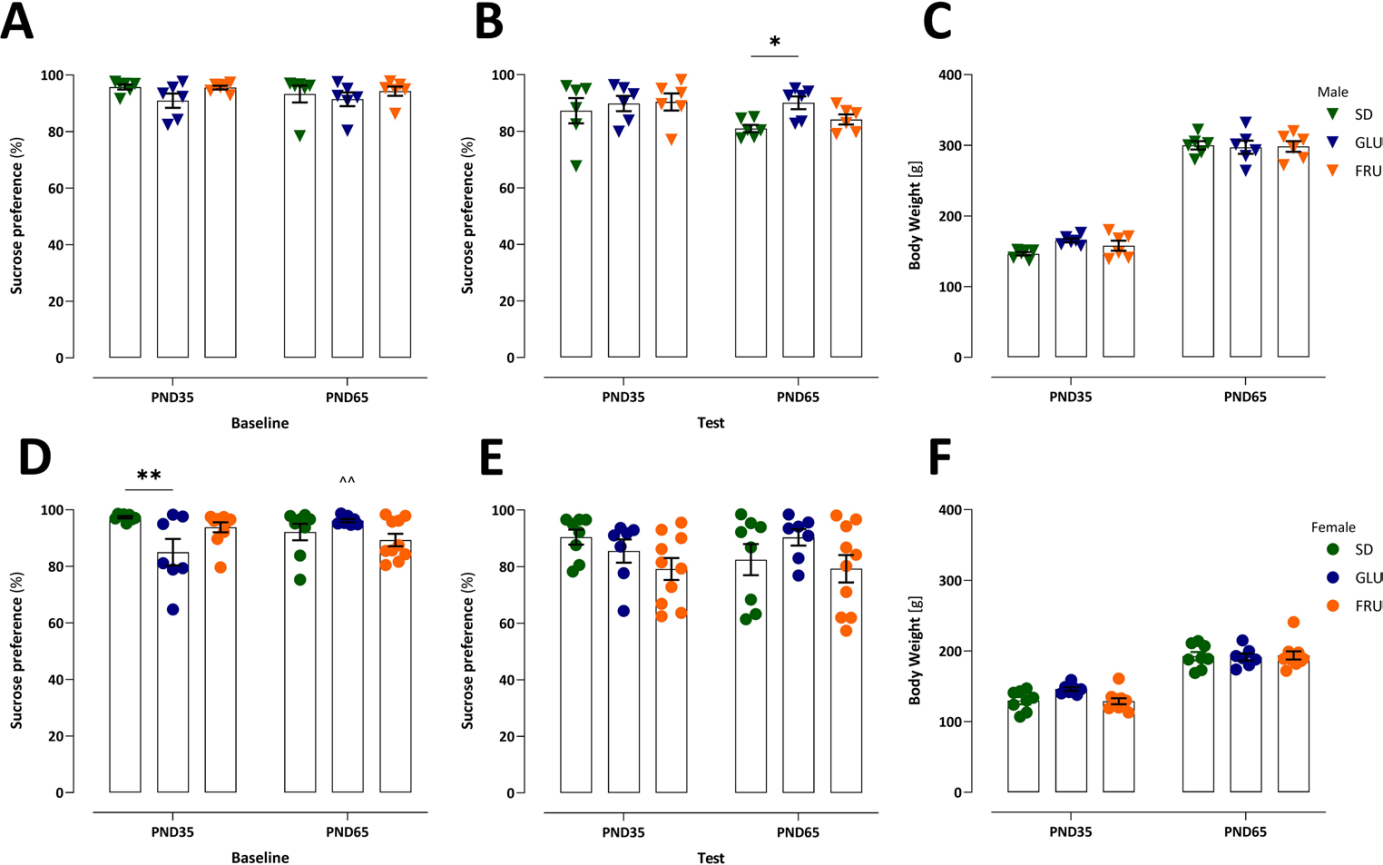
Effects of maternal glucose (GLU) or fructose (FRU) diet on sucrose preference in adolescent (PND35) and young adult (PND65) male (**A–C**) and female (**D–F**) offspring. (**A**) Sucrose preference during the baseline phase of the sucrose preference test (SPT) in male offspring. (**B**) Sucrose preference during the SPT test in male offspring. (**C**) Body weight (g) of male offspring. *n* = 6 rats per group. (**D**) Sucrose preference during the SPT baseline in female offspring. (**E**) Sucrose preference during the SPT test in female offspring. (**F**) Body weight (g) of female offspring. *n* = 8 (SD), 7 (GLU), and 10 (FRU) rats. Data are compared to the control diet (SD) groups (*p < 0.05, **p < 0.01) or age (^^*p* < 0.01), and analyzed by two-way repeated measure ANOVA followed by Dunnett’s multiple comparison test

The effect of maternal diets on stress-related anhedonia in adolescent (PND35) and young adult (PND65) male (Fig. 3A-C) and female (Fig. 3D-F) offspring was evaluated using the SPT based on a two-bottle choice paradigm and 12-hour deprivation. No interaction (F_2, 15_ = 0.23, *p* = 0.79; F_2, 15_ = 1.51, *p* = 0.25) or diet (F_2, 15_ = 2.39, *p* = 0.12; F_2, 15_ = 1.58, *p* = 0.24) effects were detected in adolescent and young adult males regarding sucrose preference during baseline (Fig. 3A) and test (Fig. 3B), respectively. However, a significant age effect was detected during the test (F_1, 15_ = 5.27, *p* = 0.04), but not during the baseline (F_1, 15_ = 0.36, *p* = 0.55). Specifically, young adult GLU males showed a 10% higher preference for sucrose compared to their corresponding SD counterparts (Fig. 3B). No significant effect of diet (F_2, 15_ = 0.53, *p* = 0.60) was observed on male body weight throughout the experiment (Fig. 3C). In adolescent and young adult females, no significant diet (F_2, 22_ = 1.37, *p* = 0.27; F_2, 22_ = 2.96, *p* = 0.07) or age (F_1, 22_ = 0.07, *p* = 0.79; F_1, 22_ = 0.07, *p* = 0.79) effects were observed in baseline (Fig. 3D) and test (Fig. 3E) measurements, respectively. Moreover, a significant interaction effect was observed in the baseline (F_2, 22_ = 7.52, *p* = 0.003), but not in the test (F_2, 22_ = 0.95, *p* = 0.40). Adolescent GLU females showed a 12% reduction in sucrose preference, which levelled off with age (Fig. 3D). Furthermore, no diet effect (F_2, 22_ = 0.69, *p* = 0.51) was significant in female body weight during the experiment (Fig. 3F). Additionally, the total volume of sucrose consumed during the experiment was measured (**Fig. ****S1**). During the two baseline days before the test, GLU adolescent and young adult males consumed 36% and 37% less sucrose, respectively, than SD rats. Conversely, only FRU young adult males exhibited a 21% increase in sucrose consumption during the test. In females, only GLU adolescent females reduced their sucrose intake by 44% during the baseline period.

### Effects of maternal monosaccharide diets on offspring glucose homeostasis

**Fig. 4:**
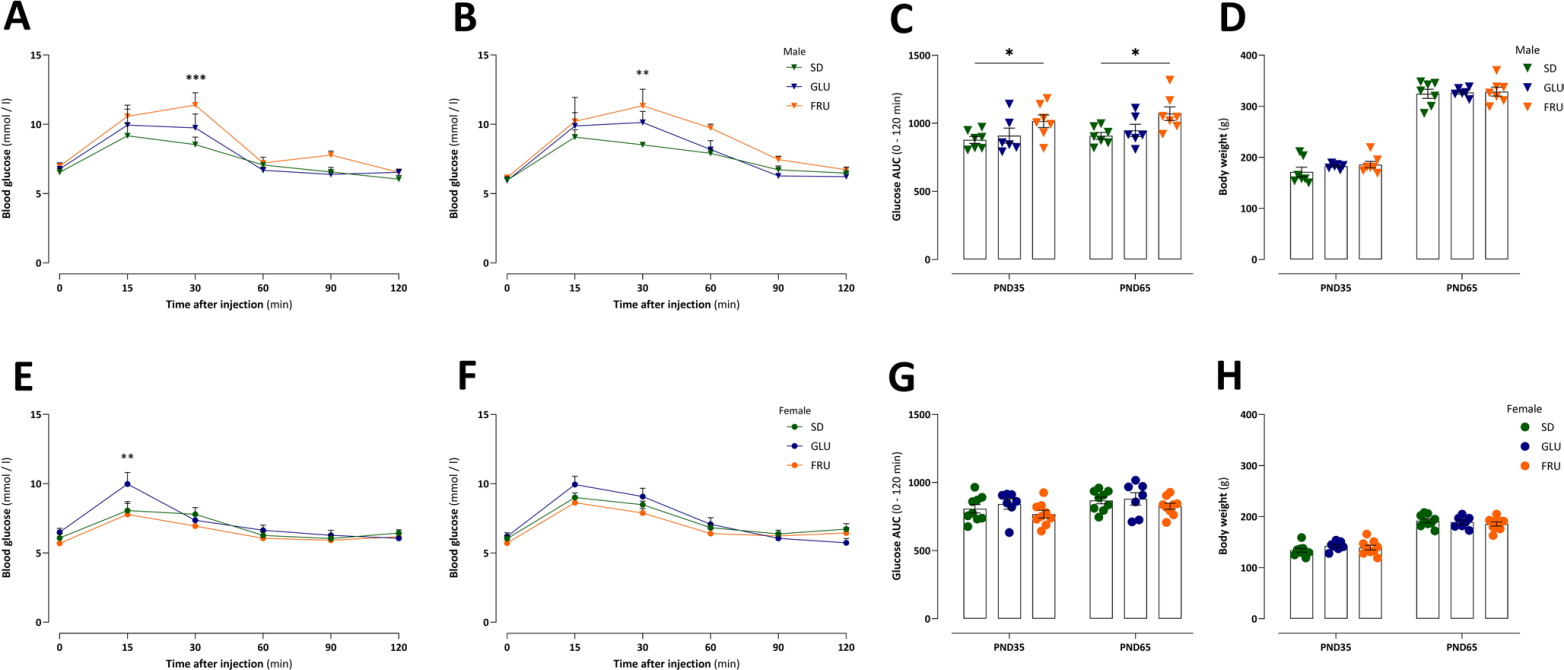
Effects of maternal glucose (GLU) or fructose (FRU) diet on glucose homeostasis in adolescent (PND35) and young adult (PND65) offspring. (**A**) Intraperitoneal glucose tolerance test (IPGTT) responses in adolescent male offspring. (**B**) IPGTT responses in young adult male offspring. (**C**) Area under the curve (AUC) of the IPGTT in adolescent and young adult male offspring. (**D**) Body weight (g) of male offspring. *n* = 7 (SD), 6 (GLU), 7 (FRU) rats. (**E**) IPGTT responses in adolescent female offspring. (**F**) IPGTT responses in young adult female offspring. (**G**) AUC of the IPGTT in adolescent and young adult female offspring. (**H**) Body weight (g) of female offspring. *n* = 11 (SD), 7 (GLU), 9 (FRU) rats. Data are compared to the control diet (SD) groups (*p < 0.05, **p < 0.01, ***p < 0.001) or time or age, and analyzed by two-way repeated measure ANOVA followed by Dunnett’s multiple comparison test

To assess glucose homeostasis, IPGTT was performed in adolescent (PND35) and young adult (PND65) offspring rats (Fig. 4). Although no diet (F_2, 17_ = 2.45, *p* = 0.12) or interaction (F_10, 85_ = 1.44, *p* = 0.17) effects were detected, the time (F_5, 85_ = 37.92, *p* < 0.0001) influenced glucose homeostasis in adolescent males. Indeed, 30 min after glucose injection, only FRU rats displayed a 25% increase in blood glucose concentration compared to SD rats (Fig. 4A). In young adult males, no interaction effect (F_10, 85_ = 0.87, *p* = 0.56) was observed; however, both diet (F_2, 17_ = 1.89, *p* = 0.03) and time (F_5, 85_ = 23.54, *p* < 0.0001) effects were significant. Similar to the adolescent pattern, young adult FRU males exhibited a 24% increase in blood glucose concentration compared to SD rats 30 min after glucose loading (Fig. 4B). In terms of total glucose AUC, strong diet effects (F_2, 17_ = 5.45, *p* = 0.01) were observed, but age (F_1, 17_ = 2.16, *p* = 0.16) and interaction (F_2, 17_ = 0.07, *p* = 0.93) effects were not. Here, FRU males showed increases of 13% and 15% in glucose AUC compared to SD males at PND35 and PND65, respectively (Fig. 4C). In body weight, no interaction (F_2, 17_ = 0.92, *p* = 0.42) or diet (F_2, 17_ = 0.51, *p* = 0.61) effects were observed; however, an age effect (F_1, 17_ = 1912.00, *p* < 0.0001) was observed during the experiment (Fig. 4D). No interaction effects (F_10, 110_ = 1.82, *p* = 0.06; F_10, 110_ = 1.74, *p* = 0.08) or diet effects (F_2, 22_ = 1.55, *p* = 0.23; F_2, 22_ = 0.99, *p* = 0.39) were observed; however, dependent changes over time (F_5, 110_ = 23.42, *p* < 0.0001; F_5, 110_ = 55.61, *p* < 0.0001) were observed in glucose homeostasis in adolescent and young adult females, respectively. After 15 min of glucose loading, only GLU females showed a 19% increase in blood glucose concentration compared to SD rats (Fig. 4E). No statistical differences were observed in young adult females, whereas this pattern was evident in adolescents (Fig. 4F). Neither interaction (F_2, 22_ = 0.30, *p* = 0.74) nor diet (F_2, 22_ = 1.23, *p* = 0.31) effects were detected; however, age effect (F_1, 22_ = 16.14, *p* = 0.0006) was noted in total glucose AUC; however, no statistically significant post hoc comparisons were found (Fig. 4G). No diet effect (F_2, 22_ = 0.20, *p* = 0.82) was observed; however, age (F_1, 22_ = 1492.00, *p* < 0.001) and interaction (F_2, 22_ = 9.57, *p* = 0.001) effects influenced body weight in female rats (Fig. 4H).

### Effects of maternal monosaccharide diets on offspring serum metabolic parameters

The effect of maternal diet on the levels of key metabolic parameters were measured in the serum of adolescent (PND28) and young adult (PND63) offspring (Table 2). In adolescent rats, no diet effects were observed in glucose (H = 5.66, *p* = 0.34, N_1, 2, 3_ = 8), HDL (H = 9.10, *p* = 0.10, N_1, 2, 3_ = 8), and LDL (H = 5.21, *p* = 0.39, N_1, 2, 3_ = 8) concentrations. Neither sex (F_1, 42_ = 3.48, *p* = 0.07) nor interaction (F_2, 42_ = 0.33, *p* = 0.72) effects were significant in triglycerides concentration. Although the effect of diet (F_2, 42_ = 4.86, *p* = 0.01) was significant, no changes between groups were detected in triglyceride concentrations. Additionally, the serum concentration of cholesterol showed no interaction (F_2, 42_ = 1.56, *p* = 0.22), but both diet (F_2, 42_ = 4.66, *p* = 0.01) and sex (F_1, 42_ = 8.12, *p* = 0.007) dependent effects were significant. Indeed, the maternal GLU diet reduced cholesterol levels in males, while SD females exhibited lower concentrations than SD males. In adolescent body weight, a significant effect of diet was observed (F_2, 42_ = 25.03, *p* < 0.0001), but neither sex (F_1, 42_ = 2.17, *p* = 0.15) nor interaction (F_2, 42_ = 1.66, *p* = 0.20) effects were significant. Indeed, GLU and FRU males and females, as adolescent offspring, weighed more than SD rats, while only FRU females had a lower body weight than their corresponding male counterparts. In young adult offspring, no significant effects of diet were observed on LDL levels (H = 9.69, *p* = 0.08, N_1, 2, 3_ = 8). Additionally, no effect of diet (F_2, 42_ = 1.65, *p* = 0.20), sex (F_1, 42_ = 0.83, *p* = 0.37), or interaction (F_2, 42_ = 0.24, *p* = 0.78) was significant in glucose concentration. Although the effect of diet on HDL levels (H = 21.34, *p* = 0.0007, N_1, 2, 3_ = 8) was significant, no changes were observed between groups. In cholesterol concentrations, only the sex (F_1, 42_ = 12.19, *p* = 0.001), but not diet (F_2, 42_ = 2.55, *p* = 0.09) and interaction (F_2, 42_ = 0.51, *p* = 0.61) effects were detected. Indeed, female offspring from the SD and FRU diets exhibited lower cholesterol levels than their male counterparts. Interestingly, both diet (F_2, 42_ = 10.55, *p* = 0.0002) and sex (F_1, 42_ = 54.27, *p* < 0.0001) effects were detected in triglyceride concentrations, with no significant interaction (F_2, 42_ = 0.71, *p* = 0.50). Maternal FRU diet significantly decreased triglyceride levels in males and females compared to the SD group. Moreover, females had lower triglyceride concentrations than males. In young adults, we observed significant effects of diet (F_2, 42_ = 9.56, *p* = 0.0004) and sex (F_1, 42_ = 1882.00, *p* < 0.0001), but no significant interaction (F_2, 42_ = 1.14, *p* = 0.33) on body weight. Only male and female young adult FRU offspring had higher body weights than SD rats; moreover, female offspring weighed less than their corresponding male counterparts.

**Table 2:** Body weight (g) and serum metabolic parameters concentrations (mg/dL) were evaluated in adolescent (PND28) and young adult (PND63) male and female offspring following maternal control (SD), glucose (GLU), or fructose (FRU) diets

	Male			Female		
	SD	GLU	FRU	SD	GLU	FRU
**PND28**						
Body weight (g)	**63.87 ± 1.48**	**72.25 ± 2.11** **	**76.50 ± 1.79 ** ****	**62.37 ± 1.72**	**73.00 ± 1.59** ***	**71.25 ± 1.09** **, **^**
Glucose (mg/dL)	159.37 ± 3.67	151.62 ± 2.84	154.62 ± 5.23	148.50 ± 4.53	152.62 ± 2.41	149.50 ± 6.23
Cholesterol (mg/dL)	115.37 ± 5.42	**101.75 ± 3.34** *	116.37 ± 2.17	**98.87 ± 3.39** **^^**	99.00 ± 3.72	108.25 ± 4.67
HDL (mg/dL)	41.87 ± 2.41	34.25 ± 1.23	38.25 ± 1.55	36.75 ± 0.86	36.50 ± 0.91	39.50 ± 1.46
LDL (mg/dL)	45.12 ± 3.95	45.37 ± 4.37	47.62 ± 1.66	39.12 ± 3.55	40.50 ± 3.03	40.37 ± 3.82
Triglycerides (mg/dL)	142.00 ± 18.71	101.87 ± 6.56	145.12 ± 13.77	115.37 ± 8.72	94.37 ± 7.62	122.62 ± 14.33
**PND63**						
Body weight (g)	**275.87 ± 2.81**	**283.37 ± 3.25**	**289.12 ± 1.74 ** **	**177.87 ± 2.57** **^^^^**	**177.00 ± 2.50** **^^^^**	**188.87 ± 3.88** *, **^^^^**
Glucose (mg/dL)	167.62 ± 9.28	153.25 ± 7.59	155.75 ± 8.53	160.00 ± 8.66	153.62 ± 6.27	146.37 ± 1.86
Cholesterol (mg/dL)	72.87 ± 3.40	69.62 ± 3.98	64.37 ± 4.38	**57.87 ± 4.81** ^	62.50 ± 5.27	**50.75 ± 1.71** ^
HDL (mg/dL)	37.75 ± 2.18	32.87 ± 1.87	32.37 ± 1.60	28.62 ± 2.48	28.62 ± 2.17	23.50 ± 0.80
LDL (mg/dL)	6.01 ± 0.54	4.34 ± 0.32	6.09 ± 0.42	7.47 ± 1.51	4.85 ± 0.68	5.46 ± 0.58
Triglycerides (mg/dL)	254.50 ± 22.42	205.25 ± 12.87	**158.00 ± 17.09** ***	**130.25 ± 20.73 ** **^^^^**	**110.87 ± 16.59 ** **^^^**	**72.00 ± 6.65** *, **^^^**

*n* = 8 rats per group. Data are compared to the SD groups (*p < 0.05, **p < 0.001, ***p < 0.001, ****p < 0.0001) or between sexes in the same diet group (^*p* < 0.05, ^^*p* < 0.01, ^^^*p* < 0.001, ^^^^*p* < 0.0001) and analyzed by two-way ANOVA or Kruskal-Wallis tests followed by tukey’s or dunn’s multiple comparison test. *HDL – high-density lipoprotein*, *LDL – low-density lipoprotein*

### Effects of maternal monosaccharide diets on offspring gene expression pattern

**Fig. 5:**
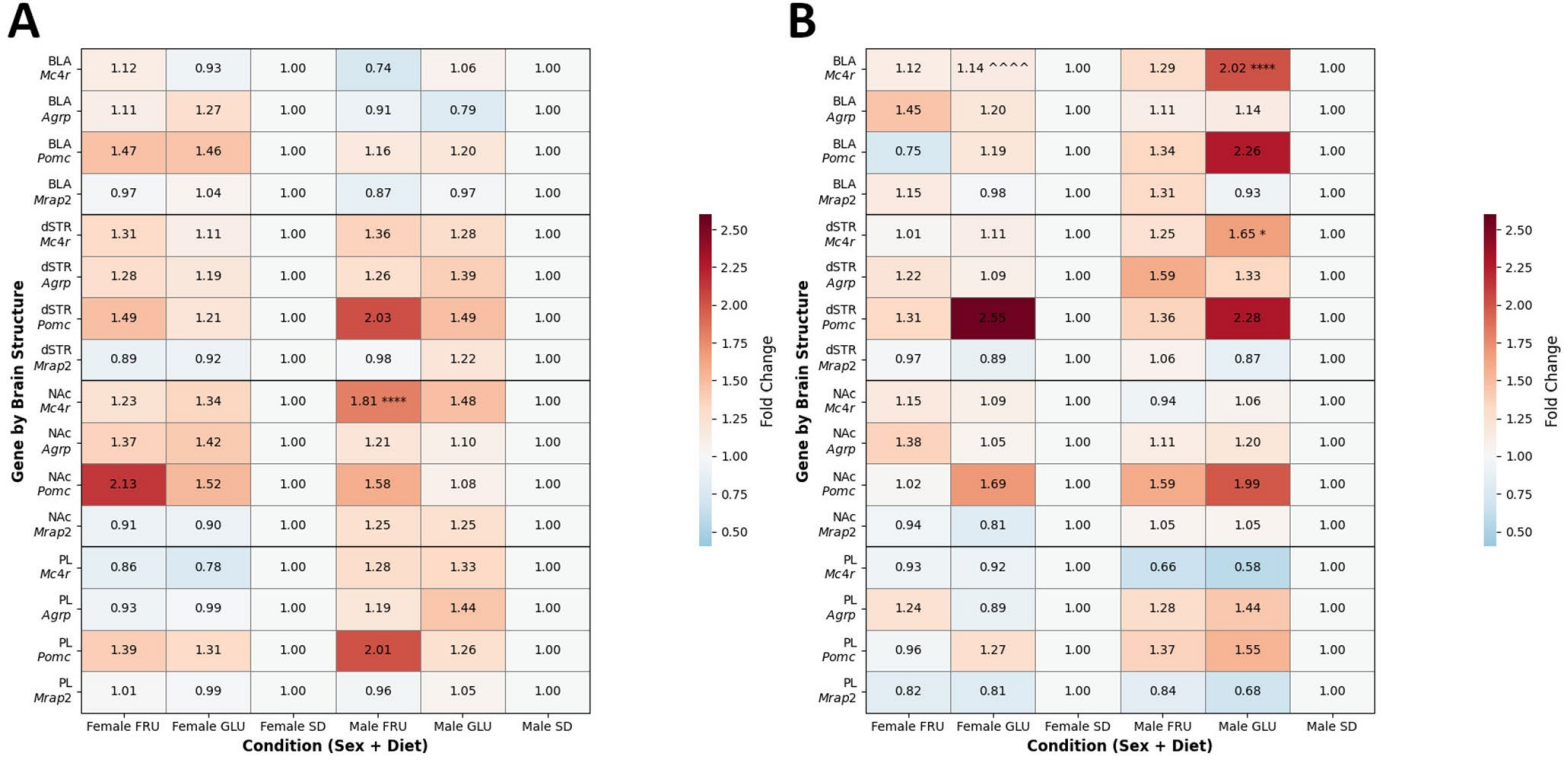
Effects of maternal glucose (GLU) or fructose (FRU) diet on offspring gene expression pattern. The heat maps represent the overall gene expression patterns expressed as mean fold change after RT-qPCR analysis of melanocortin-4 receptor (*Mc4r*), agouti-related peptide (*Agrp*), proopiomelanocortin (*Pomc*) and melanocortin-2 receptor accessory protein 2 (*Mrap2*) in male and female adolescent (**A**) and young adult (**B**) offspring brain region: the basolateral amygdala (BLA), dorsal striatum (dSTR), nucleus accumbens (NAc), and prelimbic cortex (PL). *n* = 8 rats per group. Data are compared to the control diet (SD) groups (*p < 0.05, ****p < 0.0001) or sex in the same group (^^^^*p* < 0.0001), and analyzed by three-way ANOVA followed by Tukey’s HSD multiple comparison test. The heat map scale was defined as a range from the minimum to the maximum mean fold change

To explore the impact of maternal monosaccharide diets on gene expression in male and female offspring during adolescence (PND28; Fig. 5A) and young adulthood (PND63; Fig. 5B), we quantified mRNA levels of *Agrp*, *Mc4r*, *Mrap2*, and *Pomc* via RT-qPCR in the PL, NAc, dSTR, and BLA.

In adolescents, maternal GLU or FRU diets significantly increased *Agrp* (*p* = 0.005) and *Pomc* (*p* < 0.0001) expression; however, no significant interaction effects were observed (Table S1). No significant diet effect was detected for *Mrap2* expression; however, a significant effect of sex (*p* = 0.006) and an interaction between sex and brain structure (*p* = 0.001) were observed, indicating that females exhibited lower *Mrap2* expression than males, especially in the NAc. For *Mc4r*, significant effects of maternal diet (*p* = 0.001), sex (*p* = 0.005), and brain structure (*p* < 0.0001) were observed. Both GLU and FRU maternal diets increased *Mc4r* expression, with higher levels in males compared to females. Regionally, the highest *Mc4r* expression was observed in the NAc, while the lowest was in the BLA. Significant interactions between diet and brain structure (*p* = 0.02) and sex and structure (*p* = 0.02) were noted. Specifically, the maternal FRU diet enhanced *Mc4r* expression in the NAc, and females displayed lower *Mc4r* expression than males in the PL. A significant combined effect of diet, sex, and structure (*p* = 0.04) indicated that the maternal FRU diet particularly increased *Mc4r* expression in the NAc of male offspring (Fig. 5A; Table S1).

In young adult offspring, maternal diet significantly affected *Agrp* expression (*p* < 0.0001), with both GLU and FRU diets increasing its levels; however, no significant interactions were observed (Table S1). For *Mrap2*, significant effects of diet (*p* = 0.0003), brain structure (*p* < 0.0001), and their interaction (*p* = 0.02) were noted. The maternal GLU diet significantly decreased *Pomc* expression, particularly in the PL. Regionally, *Pomc* expression was lower in the PL and higher in the BLA (Table S1). Overall, significant effects were observed for *Pomc* expression related to diet (*p* < 0.0001), sex (*p* = 0.0006), and structure (*p* = 0.0007), along with significant interactions between diet and sex (*p* = 0.04) and diet and structure (*p* = 0.003). The maternal GLU diet increased *Pomc* expression in the BLA, NAc, and dSTR. Male rats displayed higher *Pomc* expression than females, with the dSTR showing greater expression levels than the PL. Furthermore, GLU offspring exhibited higher *Pomc* expression compared to SD controls, and FRU males showed increased *Pomc* expression compared to both FRU females and SD males (Table S1). For *Mc4r* in young adults, significant effects of diet (*p* = 0.004) and brain structure (*p* < 0.0001) were observed, along with significant interactions between diet and sex (*p* = 0.04), diet and structure (*p* = 0.0002), and sex and structure (*p* = 0.0001). The maternal GLU diet significantly increased *Mc4r* expression, particularly in the BLA of males, where expression levels were higher than those in other brain regions. Moreover, the maternal GLU diet enhanced *Mc4r* expression in males compared to SD males. Significantly, effects of diet, sex, and their interaction (*p* = 0.01) indicated that the maternal GLU diet increased *Mc4r* expression in the BLA of both male and female offspring, while decreasing expression in the dSTR of males (Fig. 5B; Table S1).

### Effects of maternal monosaccharide diets on offspring MC4R protein levels

**Fig. 6:**
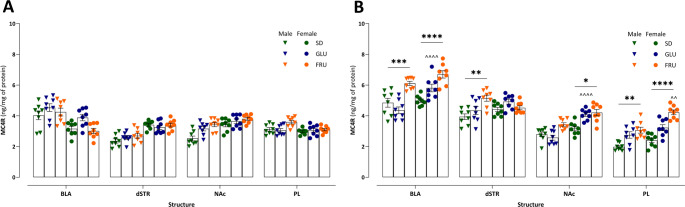
Effects of maternal glucose (GLU) or fructose (FRU) diet on melanocortin-4 receptor (MC4R) protein levels in (**A**) adolescent (PND28) and (**B**) young adult (PND63) offspring. MC4R concentrations (ng/mg protein) were measured in the synaptosomal fraction of the basolateral amygdala (BLA), dorsal striatum (dSTR), nucleus accumbens (NAc), and prelimbic cortex (PL). *n* = 8 rats per group. Data are compared to the control diet (SD) groups (*p < 0.05, **p < 0.01, ***p < 0.001, ****p < 0.0001) or sex in the same group (^^*p* < 0.01, ^^^^*p* < 0.0001), and analyzed by three-way ANOVA followed by Tukey’s HSD multiple comparison test

To evaluate the impact of maternal diets on MC4R protein levels in male and female offspring during adolescence (PND28; Fig. 6A) and young adulthood (PND63; Fig. 6B), ELISA analyses were performed on synaptosomal fractions isolated from the BLA, dSTR, NAc, and PL. In adolescents (Fig. 6A), significant main effects of diet (*p* = 0.0003) and brain structure (*p* < 0.0001) were detected; however, no significant three-way interaction among diet, sex, and structure was observed (*p* = 0.62; Table S2). Overall, the maternal FRU diet significantly increased MC4R levels in adolescent offspring, particularly in males, compared to adolescent offspring of SD rats (*p* = 0.03). An interaction between sex and structure (*p* < 0.0001) revealed that adolescent males exhibited higher MC4R levels in the BLA and lower levels in the dSTR (Table S2).

In young adults (Fig. 6B), the effects of diet (*p* < 0.0001), sex (*p* < 0.0001), and structure (*p* < 0.0001) became more pronounced (Table S2). The maternal FRU diet significantly elevated MC4R levels in young adult offspring compared to SD rats. Females exhibited higher overall MC4R levels than males did. Regionally, the highest MC4R levels were observed in the BLA of females, whereas the lowest levels were detected in the PL of males (Table S2). A significant interaction between diet and sex (*p* = 0.001) indicated that both young adult male and female FRU offspring had higher MC4R levels than their SD counterparts. Additionally, female GLU rats exhibited higher levels than their male GLU counterparts. Moreover, a significant diet-by-structure interaction (*p* = 0.0001) indicated that the FRU diet elevated MC4R levels in the BLA, NAc, and PL, while the GLU diet specifically increased levels in the PL. Additionally, a significant interaction between sex and structure (*p* = 0.003) revealed that females had higher MC4R levels than males in the BLA, NAc, and PL (Table S2). Importantly, a significant three-way interaction among diet, sex, and structure (*p* = 0.0002) underscores the complex interplay of these factors in regulating MC4R levels during young adulthood. Notably, compared to SD rats, the maternal FRU diet significantly increased MC4R protein levels in the BLA and PL of both male and female young adults, as well as in the dSTR of males and the NAc of females (Fig. 6B).

### Relationship of synaptosomal MC4R levels and metabolic parameters

**Fig. 7:**
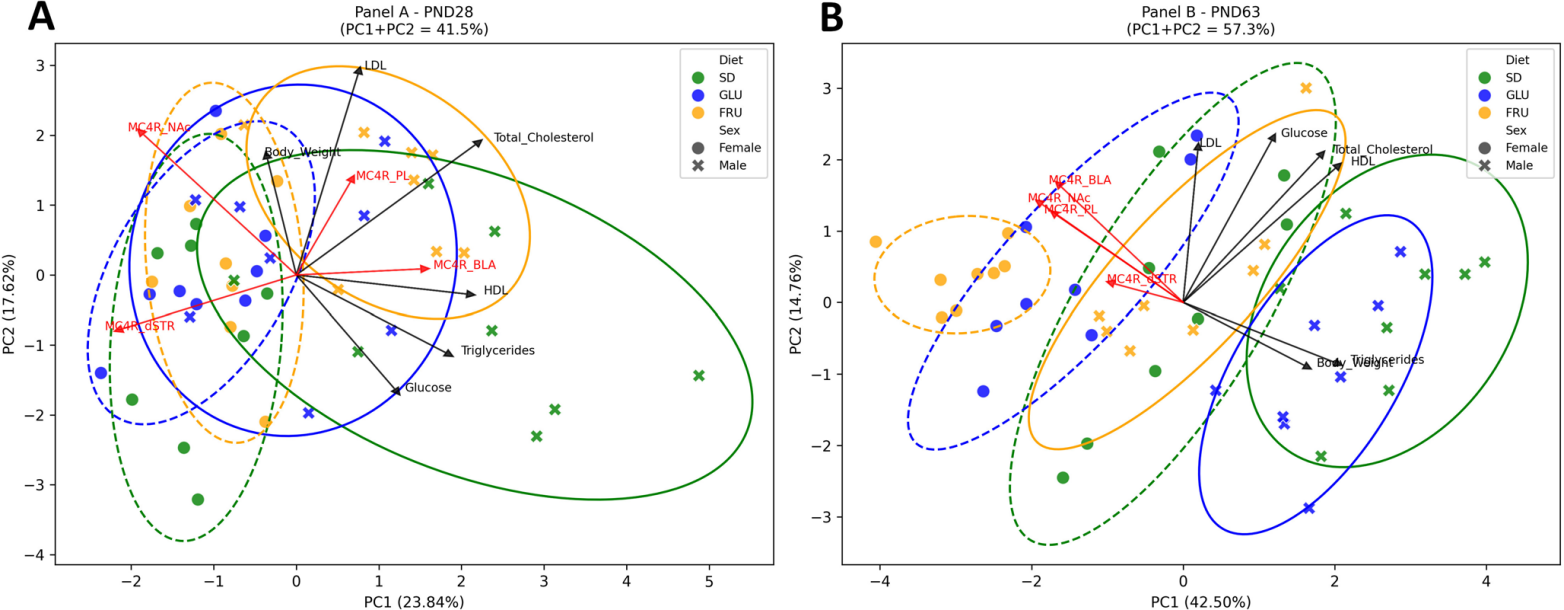
Biplots of principal component analysis (PCA) of metabolic parameters (body weight, glucose, total cholesterol, high-density lipoprotein (HDL), low-density lipoprotein (LDL), triglycerides) and synaptosomal melanocortin-4 receptor (MC4R) protein levels measured in the basolateral amygdala (BLA), dorsal striatum (dSTR), nucleus accumbens (NAc), and prelimbic cortex (PL) of (**A**) adolescent rats (PND28) and (**B**) young adult rats (PND63). Each point represents an individual animal, color-coded by maternal diet—control (SD), glucose (GLU), or fructose (FRU)—and shaped by sex (male, female). Confidence ellipses depict the 95% variability within each group. Black arrows indicate loadings of metabolic parameters, and red arrows indicate loadings of MC4R protein levels in the different brain regions on the first two principal components (PC1 and PC2). Statistical analyses included MANOVA for multivariate effects, followed by univariate ANOVAs and post hoc Tukey’s HSD tests where appropriate. *n* = 8 rats per group

PCA was conducted separately for PND28 and PND63 to explore multivariate patterns in metabolic parameters and synaptosomal MC4R protein levels in offspring subjected to varying maternal diets (Fig. 7). The adequacy of the data for PCA was confirmed by significant Bartlett’s tests for both ages (PND28: χ² = 136.19, *p* < 0.0001; PND63: χ² = 223.65, *p* < 0.0001). However, the Kaiser-Meyer-Olkin Measure of Sampling Adequacy (KMO), which assesses whether variables share enough common variance to justify PCA, was low at PND28 (0.38), suggesting weak correlations among variables and indicating caution in interpreting PCA results at this age. In contrast, the KMO at PND63 was acceptable (0.73), supporting the suitability of PCA.

At PND28 (Fig. 7A), the first two principal components accounted for 41.38% of the total variance, with PC1 explaining 23.79%, and PC2 17.59%. Variables contributing most strongly (|loading| >0.3) included total cholesterol, HDL cholesterol, triglycerides, and glucose, as well as MC4R protein levels in the dSTR, NAc, and BLA (Table S3). The biplot indicated only moderate separation between dietary groups at this developmental stage. The MANOVA for PND28 did not reveal a significant main effect of diet (Wilks’ λ = 0.90, F_4, 82_ = 1.07, *p* = 0.38), but showed a significant effect of sex (Wilks’ λ = 0.66, F_2, 41_ = 10.51, *p* = 0.0002) and a significant diet × sex interaction (Wilks’ λ = 0.76, F_4, 82_ = 2.94, *p* = 0.02), suggesting that male and female offspring responded differently to maternal dietary conditions. Subsequent univariate ANOVAs confirmed significant effects of diet on PC1 (F_2, 42_ = 5.15, *p* = 0.009, η² = 0.19) and PC2 (F_2, 42_ = 6.04, *p* = 0.005, η² = 0.22), with sex also exerting a robust influence on PC1 (F_1, 42_ = 60.62, *p* < 0.0001, η² = 0.59). However, significant differences in post hoc comparisons were observed only for PC2, where Tukey HSD testing revealed that the FRU group had significantly lower PC2 scores than the SD group (mean difference = − 1.48, *p* = 0.003). No significant pairwise differences were detected for PC1 (Table S3).

At PND63 (Fig. 7B), the first two principal components explained 57.31% of the variance, with PC1 accounting for 42.50% and PC2 for 14.81%. At young adults’ total cholesterol, HDL cholesterol, triglycerides, and glucose again showed strong loadings, alongside MC4R protein levels in the BLA, NAc, and PL regions (Table S3). The biplot at this age showed a clearer separation among dietary groups, especially for animals exposed to maternal FRU intake. MANOVA at PND63 revealed significant multivariate effects of diet (Wilks’ λ = 0.36, F_4, 82_ = 13.61, *p* < 0.0001) and sex (Wilks’ λ = 0.36, F_2, 41_ = 35.97, *p* < 0.0001), whereas the diet × sex interaction was not statistically significant (Wilks’ λ = 0.82, F_4, 82_ = 2.14, *p* = 0.08), though it approached significance. Univariate ANOVAs demonstrated a strong effect of diet (F_2, 42_ = 34.00, *p* < 0.0001, η² = 0.62) and sex (F_1, 42_ = 123.83, *p* < 0.0001, η² = 0.75) on PC1, with post hoc Tukey HSD comparisons showing significant increases in PC1 scores in the FRU group compared to the SD (mean difference = 2.71, *p* = 0.0003). No significant diet effects were observed for PC2 at PND63 (Table S3).

**Fig. 8:**
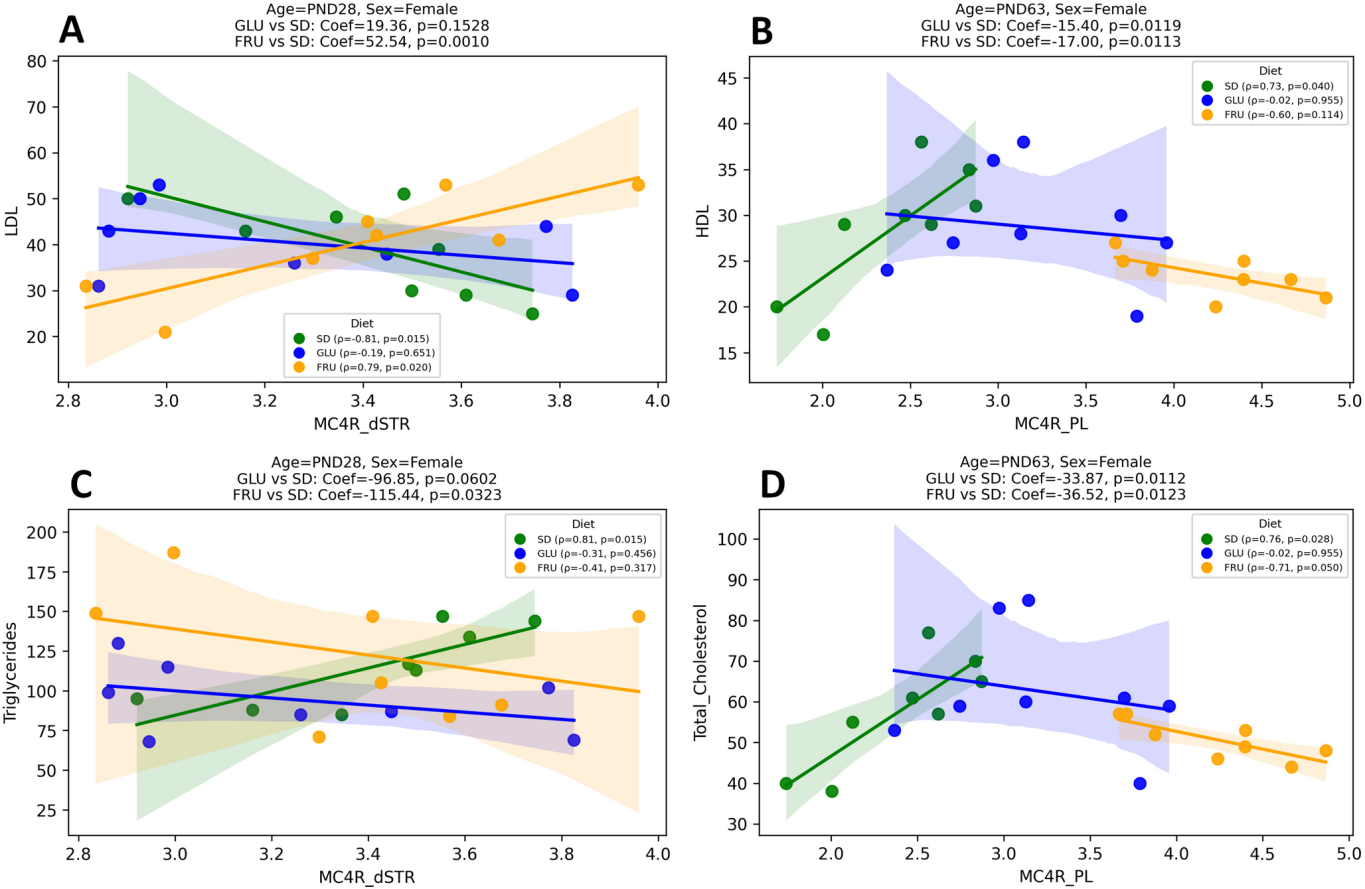
Representative associations between synaptosomal melanocortin-4 receptor (MC4R) protein levels in specific brain regions and serum lipid levels in female offspring exposed to maternal control (SD), glucose (GLU), or fructose (FRU) diets. (**A**) Correlation between MC4R in the dorsal striatum (MC4R_dSTR) and serum low-density lipoprotein (LDL) at PND28. (**B**) Correlation between MC4R in the prelimbic cortex (MC4R_PL) and serum high-density lipoprotein (HDL) at PND63. (**C**) Correlation between MC4R_dSTR and serum triglycerides at PND28. (**D**) Correlation between MC4R_PL and serum total cholesterol at PND63. *n* = 8 rats per group. Colored dots represent individual animals from SD (green), GLU (blue), and FRU (orange) groups. Solid lines show linear regression fits with shaded 95% confidence intervals. Regression coefficients and p-values for diet comparisons versus SD are displayed in each panel

Regression analyses further elucidated diet-dependent relationships between MC4R levels and metabolic parameters (Table S4). The strongest or biologically most relevant associations were selected for visualization (Fig. 8). At PND28 in females, LDL levels exhibited a strong negative correlation with MC4R in the dSTR in the SD group (Fig. 8A; Spearman ρ = − 0.81, *p* = 0.01), which reversed to a positive correlation following the FRU diet (ρ = +0.79, *p* = 0.02), with a significant difference in slopes between FRU and SD (Coef = 52.54, 95% CI [24.46, 80.62], *p* = 0.001; Cohen’s f² = 0.99). Additionally, triglycerides were significantly negatively associated with MC4R in the dSTR in females (Fig. 8C; Coef = − 115.44, *p* = 0.03; f² = 0.58). At PND63, further diet-dependent differences emerged. In females, HDL showed significant negative associations with MC4R levels following both the maternal GLU (Fig. 8B; Coef = − 15.40, *p* = 0.01; f² = 0.92) and FRU diets (Fig. 8B; Coef = − 17.00, *p* = 0.01; f² = 0.92), compared to SD. Total cholesterol was also significantly negatively related to MC4R in the PL in females following both the GLU (Fig. 8D; Coef = − 33.87, *p* = 0.01; f² = 0.84) and FRU diets (Fig. 8D; Coef = − 36.52, *p* = 0.01; f² = 0.84). Diagnostic tests showed no significant deviations from normality or heteroskedasticity in these models. No significant diet-specific associations were observed for other variables, consistent with the lower PCA loadings for those regions or parameters.

## Discussion

The present study demonstrated that the maternal monosaccharide diet significantly changed the dietary intake of the dam and energy expenditure during pregnancy and lactation. This maternal nutritional program affects the subsequent developmental trajectory of the offspring. Specifically, dams fed a GLU diet, but not those fed a FRU diet, had significantly lower body weights than those on an SD during lactation. Moreover, we observed changes in total calorie intake during lactation in GLU dams; however, FRU dams consumed more calories from monosaccharides during pregnancy and lactation. These results are consistent with our previously published data, in which we also observed decreased body weight in GLU dams, reduced total calorie intake, and increased calorie intake from sugar in both GLU and FRU dams [10]. Despite these findings, maternal GLU or FRU consumption did not affect litter size and postnatal pup weight measured per litter. However, a few previous papers using C57BL/6 mice or Sprague-Dawley rats noted that a maternal monosaccharide GLU or FRU diet lowered birth body weight when data were analyzed across all individual pups [39, 40]. It should also be considered that maternal diet may have influenced offspring development indirectly through alterations in maternal caregiving. Although no studies to date have examined maternal behavior following monosaccharide diets, evidence from high-fat diet models demonstrates that maternal diet can alter maternal–pup interactions and caregiving behaviors, thereby shaping long-term offspring outcomes [69–71].

The SPT revealed no evidence of anhedonia in the offspring following food and water deprivation stress. During baseline measurements, adolescent GLU females displayed reduced sucrose preference and lower sucrose consumption; however, no significant differences were observed during the subsequent test. Similarly, reduced sucrose consumption was noted in GLU offspring during baseline measurements, whereas young adult FRU males exhibited increased sucrose consumption during the subsequent test. These findings suggest that maternal diet al.one may not be a robust predictor of anhedonia under the current test conditions. Notably, the test was performed one hour after the reintroduction of food and water following 12 h of deprivation, which may not have been sufficient to reveal subtle anhedonic behaviors [72]. Nevertheless, our previous study demonstrated that maternal FRU diets increased anxiety-like behaviors in adolescent and young adult offspring using the elevated zero maze test. In contrast, both maternal GLU and FRU diets increased depressive-like behavior in adolescent male offspring in the forced swim test [10]. The changes in sucrose consumption observed here, particularly the increase in sucrose consumption in young adult FRU males, may reflect alterations in reward sensitivity or appetite regulation rather than classical anhedonia. Indeed, several studies have shown that maternal diets high in sugar can significantly influence reward sensitivity, neuropeptide signaling, hormonal appetite regulation, and taste preferences. For example, the maternal cafeteria diet has been reported to reduce dopamine signaling and induce epigenetic dysregulation of dopamine receptors (e.g., DRD2) in the ventral tegmental area of neonatal Wistar rat offspring. Additionally, this diet affects the NAc of adolescent rats at PND10 [73]. Moreover, a maternal high-sucrose (25%) diet has been shown to increase preference for high-sucrose and high-fat diets and to enhance motivation for sugar rewards, mediated by modulation of steroid signaling within the mesocorticolimbic system of adult male Long Evans rats [74]. Additionally, several studies have demonstrated that maternal FRU intake increases circulating leptin levels [38, 39] and leptin gene expression in rat plasma [75]. Consistently, elevated leptin levels have been reported following maternal FRU [40, 76] or GLU [40] consumption in mice. These findings suggest that maternal GLU or FRU intake may influence offspring sucrose consumption by modulating reward-related pathways. Beyond the SPT, similar diet-related effects on reward-driven behaviors were observed in other paradigms. In our previous work, young adult offspring (PND61), particularly FRU males, displayed increased novelty-seeking in the novel object recognition test [10], consistent with heightened incentive salience. Furthermore, preliminary findings on cocaine self-administration in FRU males, presented in our recent conference abstract [77], revealed greater active lever presses to obtain cocaine under increasing FR schedules but reduced intake at higher cocaine doses in the escalation paradigm.

In IPGTT measurements, we observed elevations in blood glucose homeostasis in adolescent FRU male offspring, and this pattern persisted when these animals were retested in young adulthood. Conversely, adolescent GLU female offspring showed increased blood glucose levels 15 min after glucose injection; however, this alteration was not statistically significant when retested in young adulthood. Importantly, the observed changes in glucose homeostasis were associated with higher glucose AUC only in adolescent and young adult FRU males; no significant differences in body weight were observed in either sex. These male-specific impairments in glucose tolerance may reflect greater susceptibility to the metabolic programming effects of maternal diets, potentially mediated by sex-specific differences in hormones, insulin sensitivity, or central regulation of glucose metabolism. Supporting our results, supplementation with FRU (10–20 g/kg) during pregnancy did not affect blood glucose levels or glucose AUC in the dams; however, it resulted in elevated basal blood glucose and higher AUC during oral glucose tolerance testing in 7-week-old Sprague-Dawley offspring [39]. Additionally, a maternal FRU diet consisting of 49.7% FRU increased blood glucose levels and glucose AUC in both male and female Sprague-Dawley offspring at PND87, with the AUC being higher in males [38]. Another study involving 12-week-old C57BL/6J mouse offspring examined the effects of maternal feeding with GLU (480 g/kg) or FRU (480 g/kg) during pregnancy and lactation. This study observed altered blood glucose patterns after oral glucose loading; however, no significant changes in glucose AUC were noted [40]. Maternal FRU intake has also been linked to increased fasting insulin and impaired insulin sensitivity [38, 39], while 20% HFCS supplementation induced insulin resistance via downregulation of hepatic gene expression in Sprague-Dawley rats [78]. Taken together, these findings suggest that the elevations in blood glucose observed in our offspring may reflect disruptions in glucose metabolism resulting from fetal metabolic reprogramming induced by maternal monosaccharide intake [79, 80].

In the serum lipid panel, we observed changes in cholesterol and triglyceride levels. It is well-established that the maternal diet can induce metabolic reprogramming in offspring [81]. For example, exposure to a maternal 49.7% FRU diet until PND87 elevated cholesterol and triglyceride levels in male and female Sprague–Dawley offspring [38]. In contrast, the present study observed decreased cholesterol levels in adolescent GLU males. Additionally, triglyceride levels were reduced in male and female young adult offspring exposed to maternal FRU. These findings align with reports showing that maternal supplementation with HFCS failed to significantly alter plasma cholesterol, triglycerides, HDL, or LDL, but induced slight reductions in 3- and 12-week-old albino Wistar offspring [82]. Similarly, no significant differences in triglyceride concentrations were detected in 12-week-old male and female C57BL/6J offspring following maternal GLU or FRU diets, although a slight reduction was again noted [40]. Consistently, high-FRU (444 g/kg) exposure via maternal or paternal diet in C57BL/6 mice did not affect cholesterol or triacylglycerol levels, but modest reductions were observed [76]. These discrepancies in metabolite concentrations highlight the influence of several factors, including offspring age, strain, and specific composition of the maternal diet. Beyond lipid metabolism, maternal diet modulates key hormonal regulators of fat storage and energy balance, including insulin and leptin. Although we observed increased body weight in offspring exposed to GLU or FRU diets, this effect was absent in individually housed animals during SPT and IPGTT testing, consistent with our previous observation that weight differences emerge only in group-housed offspring [10]. Notably, GLU- and FRU-exposed offspring displayed relatively low birth weights, followed by accelerated postnatal weight gain, suggesting a compensatory response to suboptimal fetal nutrition. Interestingly, this weight gain coincided with an age-dependent decrease in circulating cholesterol and triglyceride concentrations. Such a profile suggests that maternal monosaccharide diets may promote lipid redistribution, enhancing deposition into adipose tissue rather than increasing levels in circulation. Supporting this interpretation, previous studies have reported that maternal monosaccharide diets elevate fat mass and promote the accumulation of both white and brown adipose tissue in offspring [38, 40]. Taken together, these findings demonstrate that maternal GLU and FRU intake leads to peripheral dysregulation in the offspring, characterized by increased body weight and decreased lipid availability in the circulation. This pattern indicates disrupted lipid partitioning and storage underlying systemic metabolic disturbances.

This report is also the first evidence that maternal monosaccharide diets affect the offspring’s melanocortin system, particularly the MC4R, and induce changes in mesocorticolimbic brain regions. In adolescent offspring, increased *Mc4r* expression was observed in the NAc of FRU males; whereas in young adults, *Mc4r* expression was elevated by the maternal GLU diet in the BLA and dSTR. Additionally, we observed elevated MC4R protein levels in the BLA and PL of young adult offspring after maternal FRU exposure. The increased MC4R level in young adult FRU rats was sex-dependent, as evident in higher levels in the dSTR of males and the NAc of females. It is well-established that the functional knockout rat model for *Mc4r* exhibits hyperphagia, increased body weight, increased food intake [83], and elevated plasma lipid concentrations [84]. Our present study found that *Mc4r* upregulation and increased MC4R levels induced by maternal GLU or FRU diets were associated with only moderate increases in body weight, decreased triglyceride levels, and no significant changes—or even slight reductions—in other serum lipid parameters. Furthermore, FRU male offspring exhibited higher glucose AUC, accompanied by dysregulation of *Agrp*, *Pomc*, and *Mrap2* expression. A few studies have shown the impact of maternal diet on glucose AUC and MC4R signaling. For example, a maternal diet high in fat (58%) and sugar (25.6%) has been shown to increase glucose AUC and enhance *Pomc* and *Mc4r* expression in the hypothalamus of C57BL/6J mouse offspring [85]. Moreover, a maternal high-fat diet elevated glucose AUC, along with higher *Mc4r*, *Pomc*, and *Agrp* gene expression in the hypothalamus of C57BL/6J offspring [86]. Although *Agrp* and *Pomc* are predominantly expressed in hypothalamic nuclei, emerging evidence suggests that these neuropeptides or their mRNA may also be detected in mesocorticolimbic regions, either due to local expression or via projections from hypothalamic neurons [87–91]. Therefore, our detection of *Agrp* and *Pomc* transcripts in regions such as the NAc and BLA may reflect either local gene expression or the transport of mRNA in afferent fibers. This potentially indicates that maternal dietary influences extend beyond the hypothalamus and impact broader melanocortin circuits that are involved in reward and emotional processing. Relatively few studies have explored the influence of maternal diet on MC4R signaling within mesocorticolimbic circuits, which play central roles in reward processing and motivated behaviors. According to our findings, a maternal high-sucrose (44%) diet increased MC4R levels in the synaptosomal fraction of the NAc and dSTR in female Wistar offspring at PND63 [92].

Furthermore, multivariate analyses provided additional evidence that these central changes in MC4R are tightly linked to alterations in peripheral metabolism. PCA, performed separately for PND28 and PND63, revealed that key metabolic parameters—including cholesterol, HDL, triglycerides, and glucose—clustered closely with MC4R protein levels in specific brain regions. This suggests a shared variance structure between peripheral metabolic status and central melanocortin signaling. Notably, while the separation between dietary groups was moderate during adolescence, it became distinctly clearer in young adulthood, particularly under the maternal FRU diet. Regression analyses revealed specific and biologically meaningful associations: in adolescent females (PND28), LDL levels exhibited a strong negative correlation with MC4R levels in the dSTR under the SD diet; however, this relationship reversed to a positive correlation under the FRU diet, indicating a diet-dependent shift in MC4R–lipid interactions. In young adult females (PND63), HDL and total cholesterol exhibited significant negative associations with MC4R levels in the PL region under both GLU and FRU maternal diets, in contrast to the positive correlations observed under the SD condition. The MC4R signaling plays a crucial role in both central and peripheral energy metabolism [93]. Previous studies in mouse models have demonstrated that MC4R deficiency—particularly when combined with MC3R or LDL-receptor loss—exacerbates metabolic disturbances, resulting in marked hypercholesterolemia with elevated LDL and very low-density lipoprotein fractions, as well as triglycerides, thereby fostering a pro-atherogenic lipid profile [94, 95]. In contrast, our study demonstrated that maternal monosaccharide diets upregulated *Mc4r* expression and increased MC4R protein levels in specific mesocorticolimbic regions, which were associated with decreased triglyceride levels and slight reductions in serum cholesterol. Such regulation aligns with mechanistic evidence from other rodent studies, where central pharmacological activation of MC4R with the agonist melanotan II promotes lipid mobilization and reduces triglyceride storage in white adipose tissue. Conversely, the central inhibition of MC4R with the antagonist SHU9119 produces the opposite effect [96]. The use of maternal monosaccharide diets in our model enhances its translational relevance by mirroring human data linking fructose consumption to altered blood lipid profiles [97]. Reported mean daily fructose intakes in human cohorts range from 39 to 49 g/day [97], approximately 54.7 g/day [98], and from 55 to 75 g/day [99], values comparable to the FRU content in the rat diet used here. Overall, our results suggest that maternal monosaccharide diets disrupt the relationship between peripheral metabolism and central MC4R signaling, potentially leading to long-term consequences for metabolic regulation in the offspring. Although MC4R loss-of-function mutations are strongly associated with obesity, our data indicate that MC4R may exert region-specific or compensatory roles, as the maternal diet induced lasting central and peripheral disturbances despite the absence of overt obesity. These adaptations beyond the hypothalamus may contribute to long-term vulnerability to both metabolic and neurobehavioral disorders.

It is well-established that early-life fructose exposure increases anxiety- and depressive-like behaviors in rodents [8], and we recently showed that maternal GLU or FRU diets similarly induce hyperactivity, anxiety-like, and depressive-like behaviors in offspring [10]. In the present study, we confirmed that maternal monosaccharide diets affect metabolic parameters and disrupt melanocortin signaling in brain regions involved in emotional regulation, specifically the BLA, PL, NAc, and dSTR. This suggests that maternal monosaccharide intake may increase the vulnerability of offspring to emotional disturbances through MC4R pathways. Indeed, MC4R antagonists reduce immobility in the forced swim test and increase exploration in the elevated plus maze, indicating both antidepressant and anxiolytic effects [50, 51]. In humans, excessive sugar intake has been linked to obesity and metabolic syndrome [100], and MC4R mutations represent the most common monogenic cause of obesity [47, 101]. Furthermore, data from the UK Biobank suggest a role for MC4R in connecting obesity and depression [102], while a study among Iranian overweight and obese women showed an association between depression and the MC4R risk allele, particularly in individuals with unhealthy diets [103]. Despite promising preclinical findings, no MC4R antagonists have been approved for clinical use to date, likely due to concerns about metabolic side effects or a lack of receptor selectivity. However, the MC4R agonist setmelanotide was recently FDA-approved, representing a significant advancement in the treatment of rare genetic forms of obesity [64, 104]. Taken together, these findings support the notion that maternal monosaccharide diets may promote both metabolic disturbances and dysregulation of the melanocortin system, potentially contributing to emotional and metabolic disorders in offspring (Table 3).

**Table 3:** Summary of significant diet-related effects of maternal glucose (GLU) and fructose (FRU) diets on sucrose consumption (SPT), glucose area under the curve (AUC, IPGTT), *Mc4r* mRNA expression (RT-qPCR), MC4R protein levels (ELISA), body weight, serum cholesterol, and triglyceride levels in male and female offspring at adolescence (PND28/35) and young adulthood (PND63/65)

Group	GLU		FRU	
Parameter	Male	Female	Male	Female
Sucrose consumption	↔	↔	**↑** PND63	↔
AUC	↔	↔	**↑** PND35 and 65	↔
*Mc4r* mRNA expression	**↑** PND63 (BLA, dSTR)	↔	**↑** PND28 (NAc)	↔
MC4R protein levels	↔	↔	**↑** PND28 (BLA, PL) **↑** PND63 (dSTR)	**↑** PND28 (BLA, PL) **↑** PND63 (NAc)
Body weight	**↑** PND28	**↑** PND28	**↑** PND28 and 63	**↑** PND28 and 63
Cholesterol	**↓** PND28	↔	↔	↔
Triglycerides	↔	↔	**↓** PND63	**↓** PND63

Arrows indicate direction of change relative to the control diet (SD) groups: ↑ increase, ↓ decrease, ↔ no significant change. Brain regions: the basolateral amygdala (BLA), dorsal striatum (dSTR), nucleus accumbens (NAc), and prelimbic cortex (PL) showing significant differences are indicated in parentheses

To conclude, this study shows that perinatal exposure to maternal GLU and FRU diets impairs metabolic regulation and modulates central melanocortin signaling in offspring, with region- and sex-specific alterations in MC4R expression and protein levels. These changes, accompanied by metabolic shifts, may contribute to an increased risk of metabolic and mood disorders. Elucidating the functional consequences of these adaptations may inform future therapeutic approaches targeting MC4R signaling.

## Supplementary Information

Below is the link to the electronic supplementary material.

Supplementary Material 1

Supplementary Material 2

## Data Availability

No datasets were generated or analysed during the current study.

## Abbreviations

Agrp: Agouti–related peptide
AUC: Area under the curve
BLA: Basolateral amygdala
dSTR: Dorsal striatum
FRU: Maternal fructose diet
GLU: Maternal glucose diet
HDL: High–density lipoprotein
IPGTT: Intraperitoneal glucose tolerance test
LDL: Low–density lipoprotein
Mc4r: Melanocortin–4 receptor
NAc: Nucleus accumbens
PCA: Principal component analysis
PL: Prelimbic cortex
PND: Postnatal day
Pomc: Proopiomelanocortin
SD: Control diet/Maternal control diet
SPT: Sucrose preference test
