# Supplementary figures and images for: Maternal monosaccharide diets modulate melanocortin-4 receptor signaling and metabolic state in rat offspring
Source: Pharmacol Rep. 2025 Sep 16;77(6):1657–77. doi: 10.1007/s43440-025-00785-8 (PMC12647268; doi:10.1007/s43440-025-00785-8)

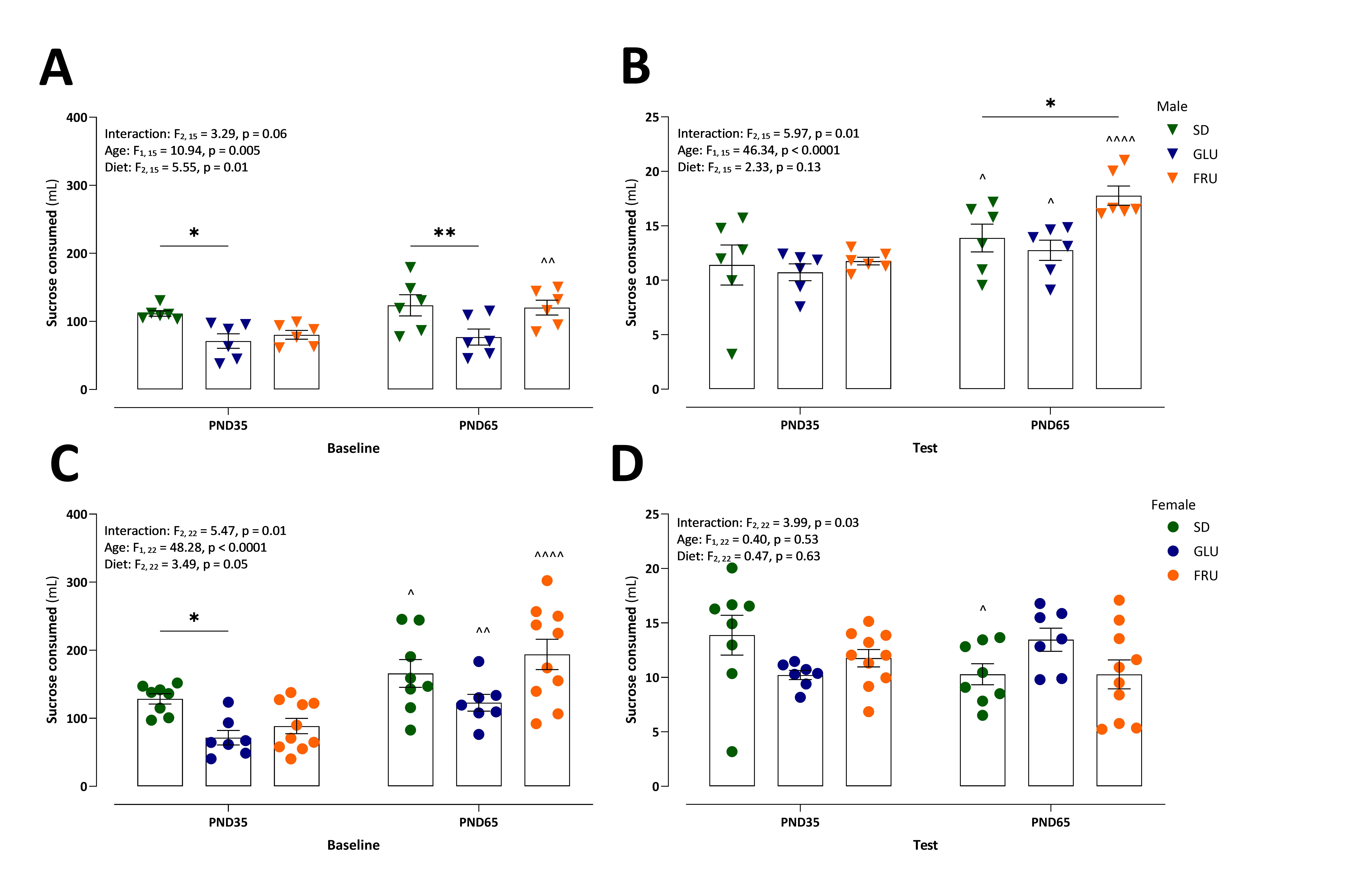

Supplement: Supplementary file 1 — Supplementary Material 1 [file 43440_2025_785_MOESM1_ESM.jpg]
