# Supplementary material for: Maternal monosaccharide diets modulate melanocortin-4 receptor signaling and metabolic state in rat offspring
Source: Pharmacol Rep. 2025 Sep 16;77(6):1657–77. doi: 10.1007/s43440-025-00785-8 (PMC12647268; doi:10.1007/s43440-025-00785-8)
Supplement: Supplementary file 2 — Supplementary Material 2 [file 43440_2025_785_MOESM2_ESM.docx]

**Fig. S1**

Effects of maternal glucose (GLU) or fructose (FRU) diet on adolescent (at PND35) and young adult (at PND65) offspring sucrose consumption (mL) during SPT. **(A)** Adolescent and young adult male offspring's sucrose consumption during the SPT baseline. (**B)** Adolescent and young adult male offspring's sucrose consumption during the SPT test. n = 6 rats per group. **(C)** Adolescent and young adult female offspring's sucrose consumption during the SPT baseline. **(D)** Adolescent and young adult female offspring's sucrose consumption during the SPT test. n = 8 (SD), 7 (GLU), 10 (FRU) rats. Data are compared to the control diet (SD) groups (*p < 0.05, **p < 0.01) or age (^p < 0.05, ^^p < 0.01, ^^^^p < 0.0001), and analyzed by two-way repeated measure ANOVA followed by Dunnett's multiple comparison test.

**TABLE S1** Corresponding to **Fig. 5 s**tatistical analysis of *Agrp*, *Mc4r*, *Mrap2*, and *Pomc* gene expression in adolescent (at PND28) and young adult (at PND63) offspring following maternal control (SD), glucose (GLU) and fructose (FRU) diet in the basolateral amygdala (BLA), dorsal striatum (dSTR), nucleus accumbens (NAc), and prelimbic cortex (PL). n = 8 rats per group. Analyzed by three-way ANOVA followed by Tukey’s HSD multiple comparison test (significant comparisons of diet x sex x structure interactions were presented in the **Fig. 5**).

| **PND** | **Gene** | **Effect** | **Sum of squares** | **df** | **F-Statistic** | **p-value** | **Effect size (**$\boldsymbol{\eta}_{\boldsymbol{p}}^{\boldsymbol{2}}$**)** | **Tukey HSD post hoc tests** |
| --- | --- | --- | --- | --- | --- | --- | --- | --- |
| **28** | ***Agrp*** | Diet | 1.40 | 2.0 | 5.48 | 0.005 | 0.06 | SD vs. GLU, p = 0.008  SD vs. FRU, p = 0.04 |
|  |  | Sex | 0.02 | 1.0 | 0.18 | 0.67 | 0.001 |  |
|  |  | Structure | 0.97 | 3.0 | 2.54 | 0.06 | 0.04 |  |
|  |  | Diet x Sex | 0.01 | 2.0 | 0.05 | 0.95 | 0.005 |  |
|  |  | Diet x Structure | 0.64 | 6.0 | 0.84 | 0.54 | 0.03 |  |
|  |  | Sex x Structure | 1.62 | 3.0 | 4.22 | 0.006 | 0.07 | - |
|  |  | Diet x Sex x Structure | 1.18 | 6.0 | 1.54 | 0.17 | 0.05 |  |
|  | ***Mc4r*** | Diet | 1.60 | 2.0 | 7.04 | 0.001 | 0.08 | SD vs. GLU, p = 0.05  SD vs. FRU, p = 0.006 |
|  |  | Sex | 0.93 | 1.0 | 8.20 | 0.004 | 0.05 | Female vs. Male, p = 0.01 |
|  |  | Structure | 3.18 | 3.0 | 9.32 | < 0.0001 | 0.14 | BLA vs. NAc, p = 0.0001  BLA vs. dSTR, p = 0.04  NAc vs. PL, p = 0.003 |
|  |  | Diet x Sex | 0.52 | 2.0 | 2.27 | 0.11 | 0.03 |  |
|  |  | Diet x Structure | 1.79 | 6.0 | 2.62 | 0.02 | 0.08 | FRU NAc vs. SD NAc, p = 0.004 |
|  |  | Sex x Structure | 1.17 | 3.0 | 3.42 | 0.02 | 0.06 | Female PL vs. Male PL, p = 0.04 |
|  |  | Diet x Sex x Structure | 1.50 | 6.0 | 2.20 | 0.04 | 0.07 | FRU Male NAc vs. SD Male NAc, p = 0.0009 |
|  | ***Mrap2*** | Diet | 0.13 | 2.0 | 1.82 | 0.16 | 0.02 |  |
|  |  | Sex | 0.28 | 1.0 | 7.77 | 0.006 | 0.04 | Female vs. Male, p = 0.009 |
|  |  | Structure | 0.16 | 3.0 | 1.47 | 0.22 | 0.02 |  |
|  |  | Diet x Sex | 0.20 | 2.0 | 2.86 | 0.06 | 0.03 |  |
|  |  | Diet x Structure | 0.16 | 6.0 | 0.74 | 0.62 | 0.02 |  |
|  |  | Sex x Structure | 0.58 | 3.0 | 5.38 | 0.001 | 0.09 | Female NAc vs. Male NAc, p = 0.001 |
|  |  | Diet x Sex x Structure | 0.34 | 6.0 | 1.57 | 0.16 | 0.05 |  |
|  | ***Pomc*** | Diet | 13.82 | 2.0 | 25.54 | < 0.0001 | 0.23 | SD vs. GLU, p = 0.003  SD vs. FRU, p < 0.0001 |
|  |  | Sex | 0.01 | 1.0 | 0.04 | 0.83 | 0.0002 |  |
|  |  | Structure | 0.85 | 3.0 | 1.04 | 0.37 | 0.02 |  |
|  |  | Diet x Sex | 0.31 | 2.0 | 0.58 | 0.56 | 0.007 |  |
|  |  | Diet x Structure | 1.87 | 6.0 | 1.15 | 0.33 | 0.040 |  |
|  |  | Sex x Structure | 3.05 | 3.0 | 3.76 | 0.01 | 0.06 | - |
|  |  | Diet x Sex x Structure | 2.29 | 6.0 | 1.41 | 0.21 | 0.05 |  |
| **63** | ***Agrp*** | Diet | 2.86 | 2.0 | 11.87 | < 0.0001 | 0.12 | SD vs. GLU, p = 0.02  SD vs. FRU, p < 0.0001 |
|  |  | Sex | 0.15 | 1.0 | 1.29 | 0.26 | 0.007 |  |
|  |  | Structure | 0.18 | 3.0 | 0.50 | 0.68 | 0.009 |  |
|  |  | Diet x Sex | 0.64 | 2.0 | 2.65 | 0.07 | 0.03 |  |
|  |  | Diet x Structure | 0.13 | 6.0 | 0.18 | 0.98 | 0.006 |  |
|  |  | Sex x Structure | 1.03 | 3.0 | 2.84 | 0.04 | 0.05 | - |
|  |  | Diet x Sex x Structure | 0.97 | 6.0 | 1.35 | 0.24 | 0.04 |  |
|  | ***Mc4r*** | Diet | 1.34 | 2.0 | 5.81 | 0.003 | 0.06 | SD vs. GLU, p = 0.02 |
|  |  | Sex | 0.32 | 1.0 | 2.74 | 0.10 | 0.02 |  |
|  |  | Structure | 4.62 | 3.0 | 13.35 | < 0.0001 | 0.19 | BLA vs. NAc, p = 0.03  BLA vs. PL, p < 0.0001  PL vs. dSTR, p = 0.0005 |
|  |  | Diet x Sex | 0.77 | 2.0 | 3.35 | 0.04 | 0.04 | GLU Male vs. SD Male, p = 0.02 |
|  |  | Diet x Structure | 3.22 | 6.0 | 4.66 | 0.0002 | 0.14 | GLU BLA vs. SD BLA, p = 0.001 |
|  |  | Sex x Structure | 2.55 | 3.0 | 7.38 | 0.0001 | 0.12 | Female BLA vs. Male BLA, p = 0.03 |
|  |  | Diet x Sex x Structure | 1.92 | 6.0 | 2.77 | 0.01 | 0.09 | GLU Female BLA vs. GLU Male BLA, p = 0.0002  GLU Male BLA vs. SD Male BLA, p < 0.0001  GLU Male dSTR vs. SD Male dSTR, p = 0.03 |
|  | ***Mrap2*** | Diet | 0.73 | 2.0 | 8.37 | 0.0003 | 0.09 | SD vs. GLU, p = 0.007 |
|  |  | Sex | 0.06 | 1.0 | 1.39 | 0.24 | 0.008 |  |
|  |  | Structure | 0.99 | 3.0 | 7.48 | < 0.0001 | 0.12 | BLA vs. PL, p < 0.0001 |
|  |  | Diet x Sex | 0.08 | 2.0 | 0.97 | 0.38 | 0.01 |  |
|  |  | Diet x Structure | 0.70 | 6.0 | 2.67 | 0.02 | 0.09 | GLU PL vs. SD PL, p = 0.04 |
|  |  | Sex x Structure | 0.13 | 3.0 | 1.06 | 0.37 | 0.02 |  |
|  |  | Diet x Sex x Structure | 0.19 | 6.0 | 0.75 | 0.61 | 0.03 |  |
|  | ***Pomc*** | Diet | 24.95 | 2.0 | 51.30 | < 0.0001 | 0.38 | SD vs. GLU, p < 0.0001 |
|  |  | Sex | 3.00 | 1.0 | 12.35 | 0.0006 | 0.07 | Female vs. Male, p = 0.009 |
|  |  | Structure | 4.34 | 3.0 | 5.95 | 0.0007 | 0.10 | PL vs. dSTR, p = 0.02 |
|  |  | Diet x Sex | 1.53 | 2.0 | 3.15 | 0.04 | 0.04 | FRU Female vs. FRU Male, p = 0.03  FRU Male vs. SD Male, p = 0.03  GLU Female vs. SD Female, p < 0.0001  GLU Male vs. SD Male, p < 0.0001 |
|  |  | Diet x Structure | 5.07 | 6.0 | 3.48 | 0.002 | 0.11 | GLU BLA vs. SD BLA, p = 0.007  GLU NAc vs. SD NAc, p = 0.0007  GLU dSTR vs. SD dSTR, p < 0.0001 |
|  |  | Sex x Structure | 2.38 | 3.0 | 3.26 | 0.02 | 0.05 | - |
|  |  | Diet x Sex x Structure | 2.01 | 6.0 | 1.38 | 0.22 | 0.05 |  |

**TABLE S2** Corresponding to **Fig. 6** statistical analysis of MC4R protein level in adolescent (at PND28) and young adult (at PND63) offspring following maternal control (SD), glucose (GLU) and fructose (FRU) diet in the basolateral amygdala (BLA), dorsal striatum (dSTR), nucleus accumbens (NAc), and prelimbic cortex (PL). n = 8 rats per group. Analyzed by three-way ANOVA followed by Tukey’s HSD multiple comparison test (significant comparisons of diet x sex x structure interactions were presented in the **Fig. 6**).

| **PND** | **Effect** | **Sum of squares** | **df** | **F-Statistic** | **p-value** | **Effect size (**$\boldsymbol{\eta}_{\boldsymbol{p}}^{\boldsymbol{2}}$**)** | **post hoc tests** |
| --- | --- | --- | --- | --- | --- | --- | --- |
| **28** | Diet | 3.28 | 2.0 | 8.43 | 0.0003 | 0.09 | SD vs. FRU, p = 0.04 |
|  | Sex | 0.21 | 1.0 | 1.10 | 0.30 | 0.006 | - |
|  | Structure | 19.41 | 3.0 | 33.19 | < 0.0001 | 0.37 | BLA vs. NAc, p = 0.0006  BLA vs. PL, p < 0.0001  BLA vs. dSTR, p < 0.0001  NAc vs. dSTR, p = 0.01 |
|  | Diet x Sex | 1.34 | 2.0 | 3.43 | 0.03 | 0.04 | FRU Male vs. SD Male, p = 0.04 |
|  | Diet x Structure | 5.07 | 6.0 | 4.34 | 0.0004 | 0.13 | - |
|  | Sex x Structure | 22.27 | 3.0 | 38.08 | < 0.0001 | 0.40 | Female BLA vs. Male BLA, p < 0.0001  Female NAc vs. Male NAc, p = 0.001  Female dSTR vs. Male dSTR, p < 0.0001 |
|  | Diet x Sex x Structure | 0.85 | 6.0 | 0.73 | 0.62 | 0.02 | - |
| **63** | Diet | 39.88 | 2.0 | 74.89 | < 0.0001 | 0.47 | SD vs. FRU, p < 0.0001 |
|  | Sex | 17.90 | 1.0 | 67.24 | < 0.0001 | 0.28 | Female vs. Male, p = 0.0008 |
|  | Structure | 188.79 | 3.0 | 236.33 | < 0.0001 | 0.81 | BLA vs. NAc, p < 0.0001  BLA vs. PL, p < 0.0001  BLA vs. dSTR, p < 0.0001  NAc vs. PL, p = 0.04  NAc vs. dSTR, p < 0.0001  PL vs. dSTR, p < 0.0001 |
|  | Diet x Sex | 3.82 | 2.0 | 7.17 | 0.001 | 0.08 | FRU Female vs. SD Female, p = 0.001  FRU Male vs. SD Male, p = 0.005  GLU Female vs. GLU Male, p = 0.009 |
|  | Diet x Structure | 7.60 | 6.0 | 4.76 | 0.0001 | 0.14 | FRU BLA vs. SD BLA, p < 0.0001  FRU NAc vs. SD NAc, p < 0.0001  FRU PL vs. SD PL, p < 0.0001  GLU PL vs. SD PL, p = 0.03 |
|  | Sex x Structure | 3.76 | 3.0 | 4.71 | 0.003 | 0.08 | Female BLA vs. Male BLA, p = 0.02  Female NAc vs. Male NAc, p = 0.001  Female PL vs. Male PL, p = 0.03 |
|  | Diet x Sex x Structure | 7.28 | 6.0 | 4.56 | 0.0002 | 0.14 | FRU Female BLA vs. SD BLA Female, p < 0.0001  FRU Male BLA vs. SD Male BLA, p = 0.0006  FRU Female NAc vs. SD Female NAc, p = 0.02  FRU Female PL vs. FRU Male PL, p = 0.003  FRU Female PL vs. SD Female PL, p < 0.0001  FRU Male PL vs. SD Male PL, p = 0.01  FRU Male dSTR vs. SD Male dSTR, p = 0.001  GLU Female BLA vs. GLU Male BLA, p < 0.0001  GLU Female NAc vs. GLU Male NAc, p < 0.0001 |

**TABLE S3** Corresponding to **Fig. 7** statistical analysis of principal component loadings and results of univariate ANOVAs for the first two principal components (PC1 and PC2) of metabolic parameters and synaptosomal MC4R protein levels in adolescent (at PND28) and young adult (at PND63) offspring following maternal control (SD), glucose (GLU) and fructose (FRU) diet in the basolateral amygdala (BLA), dorsal striatum (dSTR), nucleus accumbens (NAc), and prelimbic cortex (PL). Loadings indicate the contribution of each variable to PC1 and PC2. n = 8 rats per group. Analyzed by two-way ANOVA followed by Tukey’s HSD multiple comparison test.

| **PND28** | **PC1** | **PC2** | **abs_PC1** | **abs_PC2** | **PC PND28** | **Effect** | **Sum of squares** | **df** | **F-Statistic** | **p-value** | **Effect size (**$\boldsymbol{\eta}_{\boldsymbol{p}}^{\boldsymbol{2}}$**)** | **post hoc tests** |
| --- | --- | --- | --- | --- | --- | --- | --- | --- | --- | --- | --- | --- |
| **Body_weight** | -0.07 | 0.33 | 0.07 | 0.33 | **PC1** | **Diet** | 9.49 | 2 | 5.15 | 0.01 | 0.20 | - |
| **Glucose** | 0.24 | -0.33 | 0.24 | 0.33 |  | **Sex** | 55.83 | 1 | 60.62 | < 0.0001 | 0.59 | Female vs. Male. p < 0.0001 |
| **Total_cholesterol** | 0.43 | 0.37 | 0.43 | 0.37 |  | **Diet x Sex** | 10.41 | 2 | 5.65 | 0.007 | 0.21 | GLU Male vs. SD Male, p = 0.005.  FRU Male vs. FRU Female, p = 0.002.  SD Male vs. SD Female, p < 0.0001 |
| **HDL** | 0.41 | -0.05 | 0.41 | 0.05 | **PC2** | **Diet** | 17.93 | 2 | 6.04 | 0.005 | 0.22 | SD vs. FRU. p = 0.003 |
| **LDL** | 0.15 | 0.58 | 0.15 | 0.58 |  | **Sex** | 2.48 | 1 | 1.6 | 0.20 | 0.04 | - |
| **Triglycerides** | 0.36 | -0.22 | 0.36 | 0.22 |  | **Diet x Sex** | 1.82 | 2 | 0.61 | 0.55 | 0.03 | - |
| **MC4R_BLA** | 0.30 | 0.02 | 0.30 | 0.02 |  | | | | | | | |
| **MC4R_dSTR** | -0.42 | -0.15 | 0.42 | 0.15 |  |  |  |  |  |  |  |  |
| **MC4R_NAc** | -0.37 | 0.40 | 0.37 | 0.40 |  |  |  |  |  |  |  |  |
| **MC4R_PL** | 0.13 | 0.27 | 0.13 | 0.27 |  |  |  |  |  |  |  |  |
| **PND63** | PC1 | PC2 | abs_PC1 | abs_PC2 | **PC PND63** | **Effect** | **Sum of squares** | **df** | **F-Statistic** | **p-value** | **Effect size (**$\boldsymbol{\eta}_{\boldsymbol{p}}^{\boldsymbol{2}}$**)** | **post hoc tests** |
| **Body_weight** | 0.32 | -0.17 | 0.32 | 0.17 | **PC1** | **Diet** | 59.25 | 2 | 34.00 | < 0.0001 | 0.62 | SD vs. FRU. p = 0.0003 |
| **Glucose** | 0.23 | 0.45 | 0.23 | 0.45 |  | **Sex** | 107.88 | 1 | 123.83 | < 0.0001 | 0.75 | Female vs. Male. p < 0.0001 |
| **Total_cholesterol** | 0.36 | 0.41 | 0.36 | 0.41 |  | **Diet x Sex** | 0.28 | 2 | 0.16 | 0.85 | 0.008 | - |
| **HDL** | 0.40 | 0.38 | 0.40 | 0.38 | **PC2** | **Diet** | 3.40 | 2 | 1.21 | 0.31 | 0.05 | - |
| **LDL** | 0.04 | 0.43 | 0.04 | 0.43 |  | **Sex** | 3.50 | 1 | 2.49 | 0.12 | 0.06 | - |
| **Triglycerides** | 0.40 | -0.17 | 0.40 | 0.17 |  | **Diet x Sex** | 4.99 | 2 | 1.78 | 0.18 | 0.08 | - |
| **MC4R_BLA** | -0.32 | 0.32 | 0.32 | 0.32 |  | | | | | | | |
| **MC4R_dSTR** | -0.18 | 0.05 | 0.18 | 0.05 |  |  |  |  |  |  |  |  |
| **MC4R_NAc** | -0.37 | 0.27 | 0.37 | 0.27 |  |  |  |  |  |  |  |  |
| **MC4R_PL** | -0.33 | 0.24 | 0.33 | 0.24 |  |  |  |  |  |  |  |  |

**TABLE S4** Corresponding to **Fig. 8** statistical analysis of significant regression models examining associations between synaptosomal MC4R protein levels in adolescent (at PND28) and young adult (at PND63) offspring following maternal control (SD), glucose (GLU) and fructose (FRU) diet in the basolateral amygdala (BLA), dorsal striatum (dSTR), nucleus accumbens (NAc), and prelimbic cortex (PL) and metabolic parameters. n = 8 rats per group. Separate models were calculated for males and females. The table reports global R² values, effect sizes (Cohen’s f²), regression coefficients (β) for diet-specific interactions (GLU vs. SD and FRU vs. SD) presented as β ± SE [95% CI], p-values, and diagnostic test results (Shapiro–Wilk (Sh_p) for normality, Breusch–Pagan (BP_p) for homoscedasticity). Only models with significant diet-specific interactions (p < 0.05) are presented.

| **PND** | Sex | Region | Parameter | R^2^ | f^2^ | GLU vs SD (Coef [95% CI], p) | FRU vs SD (Coef [95% CI], p) | Sh_p | BP_p |
| --- | --- | --- | --- | --- | --- | --- | --- | --- | --- |
| **28** | Female | dSTR | LDL | 0.50 | 0.99 | 19.36 ± 12.97 [-7.88, 46.60], 0.15 | 52.54 ± 13.36 [24.46, 80.62], 0.0009 | 0.90 | 0.75 |
|  | Female | dSTR | Triglycerides | 0.37 | 0.58 | -96.85 ± 48.29 [-198.31, 4.61], 0.06 | -115.44 ± 49.77 [-220.00, -10.87], 0.03 | 0.93 | 0.14 |
|  | Female | PL | HDL | 0.45 | 0.80 | -4.59 ± 6.42 [-18.08, 8.89], 0.48 | -16.47 ± 7.38 [-31.98, -0.97], 0.04 | 0.98 | 0.89 |
|  | Male | BLA | LDL | 0.26 | 0.34 | 14.80 ± 6.74 [0.64, 28.96], 0.04 | 9.44 ± 6.69 [-4.61, 23.48], 0.17 | 0.97 | 0.14 |
|  | Male | BLA | Triglycerides | 0.45 | 0.81 | -54.97 ± 25.76 [-109.09, -0.84], 0.04 | -17.36 ± 25.57 [-71.08, 36.35], 0.50 | 0.65 | 0.14 |
| **63** | Female | NAc | Triglycerides | 0.57 | 1.32 | -182.60 ± 51.78 [-291.40, -73.81], 0.002 | -45.24 ± 41.15 [-131.70, 41.22], 0.29 | 0.02 | 0.24 |
|  | Female | PL | Total_cholesterol | 0.46 | 0.84 | -33.86 ± 11.98 [-59.04, -8.69], 0.01 | -36.52 ± 13.12 [-64.09, -8.95], 0.01 | 0.08 | 0.17 |
|  | Female | PL | HDL | 0.48 | 0.92 | -15.40 ± 5.50 [-26.95, -3.84], 0.01 | -16.99 ± 6.02 [-29.65, -4.34], 0.01 | 0.52 | 0.32 |
|  | Female | PL | LDL | 0.44 | 0.79 | -8.44 ± 2.89 [-14.52, -2.35], 0.01 | -6.86 ± 3.17 [-13.52, -0.20], 0.04 | 0.10 | 0.05 |
